# Biological Evaluation and SAR Exploration of Bile Acid–Dihydroartemisinin Hybrids as Potential Anticancer Agents for Colorectal Cancer

**DOI:** 10.3390/biom16010177

**Published:** 2026-01-22

**Authors:** Daniela Perrone, Elisabetta Melloni, Lorenzo Gnudi, Fabio Casciano, Elena Pozza, Francesca Bompan, Paola Secchiero, Elena Marchesi, Maria Luisa Navacchia

**Affiliations:** 1 Department of Environmental and Prevention Sciences, University of Ferrara, 44121 Ferrara, Italy; prd@unife.it (D.P.); lorenzo.gnudi@unife.it (L.G.); francesca.bompan@unife.it (F.B.); 2 Department of Translational Medicine and LTTA Centre, University of Ferrara, 44121 Ferrara, Italy; elisabetta.melloni@unife.it (E.M.); paola.secchiero@unife.it (P.S.); 3 Department of Environmental and Prevention Sciences and LTTA Centre, University of Ferrara, 44121 Ferrara, Italy; 4 Department of Translational Medicine, University of Ferrara, 44121 Ferrara, Italy; pzzlne1@unife.it; 5 Department of Chemical, Pharmaceutical and Agricultural Sciences, University of Ferrara, 44121 Ferrara, Italy; 6 Institute for Organic Synthesis and Photoreactivity (ISOF), National Research Council of Italy (CNR), 40129 Bologna, Italy

**Keywords:** dihydroartemisinin, molecular hybridization, colorectal cancer, HCT116 cancer cells, ursodeoxycholic bile acid, chenodeoxycholic acid, click chemistry, structure-activity relationship (SAR), bile acid hybrids, biomolecules

## Abstract

Dihydroartemisinin (DHA), a first-line treatment for uncomplicated malaria, has demonstrated antitumor activity against a variety of human cancers, emphasizing its potential for repurposing as an anticancer agent. However, its short half-life and poor bioavailability hinder its application in cancer therapy. We previously demonstrated that the molecular hybridization of DHA with bile acids (BAs) enhances its anticancer activity by improving stability and reducing toxicity. Based on this rationale, here, we designed and synthesized a library of DHA-based hybrids through conjugation with ursodeoxycholic and chenodeoxycholic bile acids. Different conjugation sites and both cleavable and non-cleavable linkages were explored to enable a comprehensive structure–activity relationship analysis. The resulting BA-DHA hybrids were evaluated in vitro for their anticancer activity against HCT116 and RKO colorectal cancer cell lines. As a result of the synergistic effect of the linker type and conjugation site, the BA-DHA hybrids synthesized via click chemistry emerged as the most active compounds in both cell lines, displaying 2- to 20-fold higher activity than the parent DHA. Mechanistic investigations further revealed that the click-derived BA-DHA hybrids possess enhanced anticancer activity and antimetastatic potential, achieving comparable or even superior efficacy to the parent compound at markedly lower concentrations.

## 1. Introduction

Colorectal cancer (CRC) is one of the most common malignancies worldwide, accounting for over 1.9 million new cases and more than 900,000 deaths annually [1]. Gender-specific statistics indicate CRC as the second leading cause of cancer-associated deaths in women and the third-highest common disease in men. In recent years, a shift in CRC epidemiology has been observed, with a rising incidence of early-onset cases, particularly among people aged around 50 [2].

Widespread screening programs and enhanced early detection have increased curative surgical outcomes for early-stage CRC [3]. However, substantial inter-country variability in screening programs results in advanced-stage diagnosis for many patients, necessitating systemic therapies including targeted agents, immunotherapy, and chemotherapy. Despite advances in CRC treatments over the past two decades, the overall five-year survival rate remains around 50% [4]. Indeed, traditional chemotherapeutic agents effectively inhibit tumor growth and invasion, but they can also negatively impact the tumor microenvironment [5]. Furthermore, drug resistance remains a major challenge, limiting the effectiveness of therapy in patients with metastatic CRC [6] and contributing to a decrease in five-year survival rate below 15% [7]. Therefore, the development of new and effective treatments for CRC remains an urgent issue.

Multifactorial diseases, such as cancer, require treatments that can modulate multiple targets simultaneously, necessitating polypharmacological strategies. This approach can lead to more effective and long-lasting therapies compared to traditional ‘one drug, one target’ methods, and it can also help overcome issues like drug resistance. Molecular hybridization (MH), which involves the synthesis of a single multitarget molecule by conjugating two or more pharmacophoric units through a covalent bond, either stable or labile under physiological conditions, can be considered a modern form of polypharmacology.

Recent advancements in MH and conjugation strategies, including the design of hybrids based on natural products (NPs) as well as biomolecules, for instance, small molecule–antibody conjugates, and peptide–natural product hybrids, have demonstrated improved target specificity, binding affinity, and in vivo efficacy for the new hybrid compounds when compared to their parent scaffolds [8].

**Figure 1:**
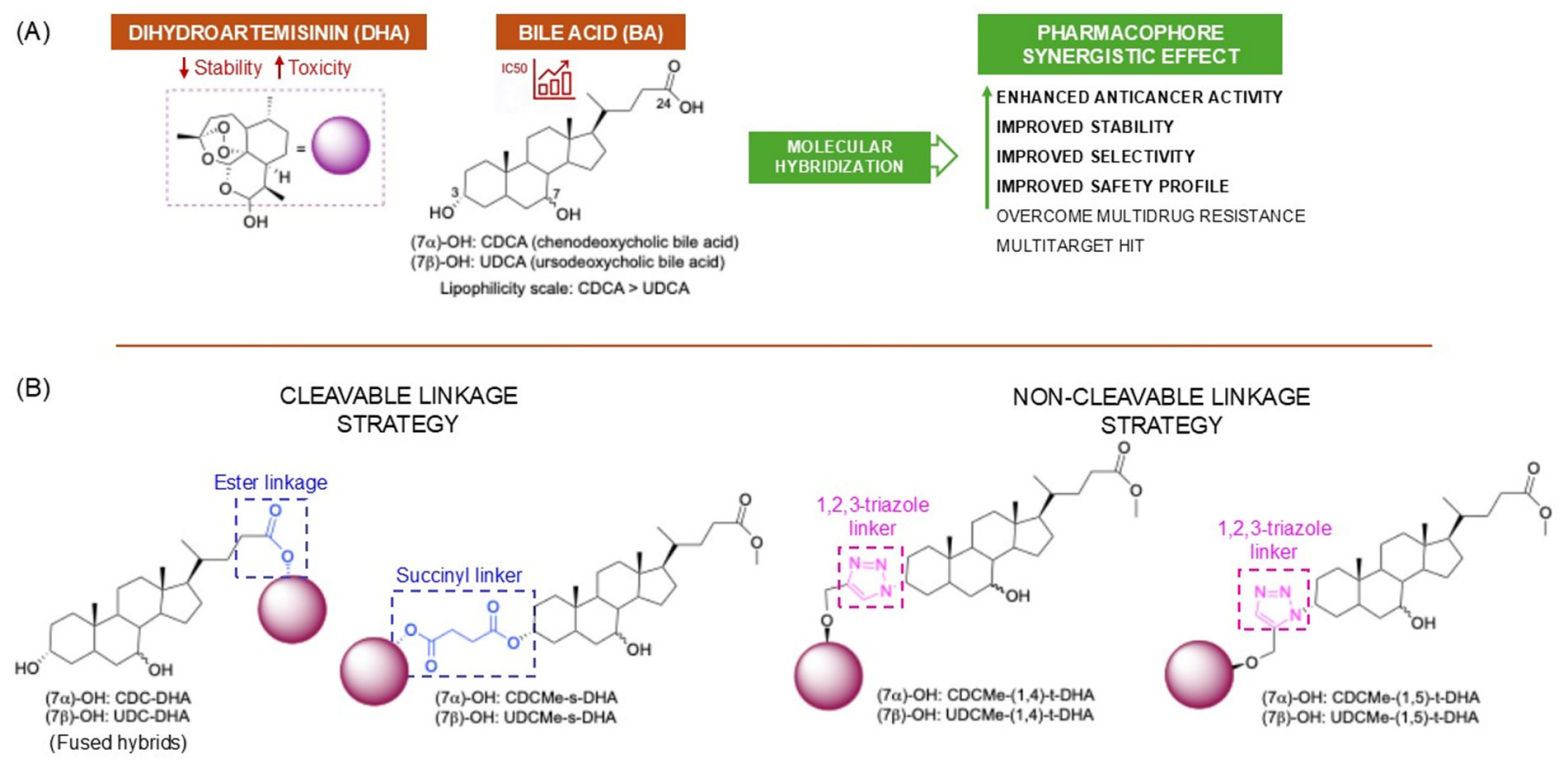
(**A**) Molecular structures of dihydroartemisinin (DHA), selected bile acids (BAs), and advantages of molecular hybridization; (**B**) molecular structures of BA-DHA hybrids considered.

Among NPs, dihydroartemisinin (DHA) (Figure 1A), a semisynthetic derivative of *Artemisia annua*, best known as a first-line treatment for uncomplicated malaria, is characterized by a broad range of biological activities, including significant anticancer effects [9]. DHA anticancer activity has been well-established in several cancer cells over the last decade, and many studies have been devoted to the disclosure of the anticancer mechanisms of DHA, which include the induction of cell cycle arrest, apoptotic cell death, autophagy, and ferroptosis, as well as the inhibition of angiogenesis, cancer cell proliferation, metastasis, and cell invasion [10,11]. In particular, DHA has been reported to reduce the viability of HCT116 CRC cells in a concentration- and time-dependent manner [12]. In vivo experiments also showed that DHA exhibits remarkable antitumor activity in CRC [13]. Despite the potential of DHA as an anticancer drug, its application in chemotherapy is limited by poor stability and low water solubility, which reduces its bioavailability and, consequently, restricts its potential broader pharmacological applications [10]. In this light, the MH of DHA with other pharmacophores represents a valuable strategy, not only to overcome its unfavorable pharmacokinetic characteristics but also to enable combination therapy within a single multifunctional agent. Such an approach can enhance both activity and selectivity, while potentially minimizing side effects and reducing drug resistance compared to traditional treatments (Figure 1A) [14,15].

Bile acids (BAs) are biomolecules derived from cholesterol metabolism, primarily synthesized in the liver and stored in the gallbladder until needed for digestion [16,17]. Due to their biocompatibility and favorable toxicity profiles [18], bile acids have attracted significant attention in the field of drug delivery. Their unique dual function as drug solubilizers and permeation enhancers makes them particularly valuable for improving the bioavailability of drugs with poor aqueous solubility or low membrane permeability [19]. In addition, bile acids can overcome gastrointestinal barriers and facilitate the carrier-mediated absorption of drugs that are complexed or conjugated with them. Beyond their conventional role as biosurfactants, BAs are increasingly recognized as signaling molecules with hormone-like functions [20,21].

In recent years, several studies have demonstrated that natural BAs can exert anticancer activity in various cancer cell types [22], including CRC [23,24,25,26,27,28,29], by inhibiting cancer cell proliferation and cell invasion/migration. The hydrophobicity of BAs, closely linked to their number of hydroxyl groups and orientation, is crucial for their biological effects [30,31,32]. Moreover, the concentration and type of bile acids, as well as the specific cancer involved, are the main factors influencing clinical outcomes [33]. However, the antitumor effects of BAs are observed only at concentrations far exceeding those compatible with pharmacological application [22,34]. Nevertheless, the unique chemical features of BAs, including their distinctive amphiphilic nature, well-defined stereochemistry, and modifiable functional groups, make them highly attractive scaffolds that have been extensively utilized in MH strategies [22]. A common and efficient synthetic approach in BA-based MH involves condensation reactions leading to the formation of prodrug conjugates. Notably, the C24 carboxylic acid group of bile acids (Figure 1A) can readily react with amino or hydroxyl functional groups, forming amide or ester derivatives, respectively. These conjugates can be selectively hydrolyzed by specific enzymes, allowing for controlled drug release under physiological conditions. On the other hand, the click reaction known as the copper-catalyzed azide–alkyne cycloaddition (CuAAC), which yields a 1,2,3-triazole linker (Figure 1B), represents a highly attractive strategy in MH for generating non-cleavable linkages. It has also been successfully employed to synthesize BA-based hybrids [35], offering robust chemical stability and structural versatility.

Over the past decade, BAs have been conjugated with a wide range of natural and synthetic bioactive compounds, including established anticancer drugs, to enhance therapeutic efficacy and selectivity [36,37].

Our research group has made significant contributions to the design and synthesis of BA conjugates with a variety of biologically relevant molecules. In particular, the MH of DHA with a selection of BAs has been successfully explored in leukemia [35,38] and hepatocellular carcinoma cell lines [38,39,40]. From our previous studies, we observed that the administration of free DHA in combination with selected BAs at low concentration was considerably less effective in cancer cells than the corresponding DHA-BA hybrids, indicating that the covalent linkage is essential [38,39,40].

In this work, we report the anticancer evaluation of two sets of BA-DHA hybrids, obtained through the conjugation of DHA with ursodeoxycholic bile acid (UDCA) and chenodeoxycholic bile acid (CDCA), respectively, targeting CRC (Figure 1A,B).

The fused hybrids UDC-DHA [38] and CDC-DHA [38] were obtained by directly exploiting functional groups already present in the parent pharmacophores (Figure 1B). The linked hybrids, UDCMe-s-DHA [41] and CDCMe-s-DHA [38], were synthesized by introducing a cleavable spacer unit not originally present in either pharmacophore (Figure 1B). Additionally, hybrids UDCMe-(1,4)-t-DHA [38,41] and UDCMe-(1,5)-t-DHA, as well as CDCMe-(1,4)-t-DHA and CDCMe-(1,5)-t-DHA, were prepared via click chemistry, through the formation of 1,2,3-triazole non-cleavable bioisosteric linkers (Figure 1B). Among these, UDCMe-(1,5)-t-DHA, as well as CDCMe-(1,4)-t-DHA and CDCMe-(1,5)-t-DHA, are reported here for the first time and were rationally designed to complete the library of BA-DHA hybrids, enabling a more comprehensive structure–activity relationship (SAR) analysis.

## 2. Materials and Methods

### 2.1. Synthesis and Characterization

#### 2.1.1. General

Commercial dihydroartemisinin (DHA) was purchased from Carbosynth (Compton, Berkshire, UK). Ursodeoxycholic bile acid (UDCA) and chenodeoxycholic acid (CDCA) were kindly furnished by ICE SpA (Reggio Emilia, Italy). Cp*RuCl(PPh_3_)_2_ was purchased from Sigma-Aldrich (St. Louis, MO, USA); copper sulfate pentahydrate (CuSO_4_·5H_2_O) and sodium ascorbate were purchased from Fluka (Buchs, Switzerland). All the reagents and solvents were used without further purification. The reactions were monitored by TLC on precoated silica gel F254 plates (thickness, 0.25 mm; Merck Life Science S. r. l., Milano, Italy) and developed with a phosphomolybdic acid solution. Flash column chromatography was performed on silica gel (60 Å, 230–400 mesh). NMR spectra were recorded with a Varian Mercury 400 MHz instrument in the stated solvent. Mass spectra were recorded in positive mode by direct infusion on an Ultivo Triple Quadrupole LC/MS (Agilent Technologies, Santa Clara, CA, USA) with Agilent Jet Stream ESI. Data were acquired with MassHunter Acquisition and processed in MassHunter Qualitative Analysis. Samples were dissolved in a mixture of CH_3_CN:HCOOH 0.1% in uH_2_O *v*/*v* 1:1. Elemental analyses (C, H, N) were performed on a PerkinElmer 2400 microanalyzer instrument. Samples were dried in vacuo prior to analysis; analytical values were within ±0.4% of the theoretical values.

#### 2.1.2. General Procedure for Copper-Catalyzed Azide–Alkyne Cycloaddition (CuAAC) Reactions

CuAAC reactions were carried out as previously reported [41]. Briefly, DHA-Alk (2.70 mmol) and 2.40 mmol of N_3_-UDCMe [41] or N_3_-CDCMe [42] were dissolved in 22 mL of CH_2_Cl_2_/H_2_O/CH_3_CN (1:1:0.1) solution. To this mixture, 0.73 mL of a 0.5 M solution of sodium ascorbate and 1.21 mL of a 0.1 M CuSO_4_ solution were added under a nitrogen atmosphere. The reaction mixture was stirred at room temperature for 1 h, then the solvent was removed under vacuum. The crude product was purified by flash chromatography.

#### 2.1.3. Characterization of Methyl (4*R*)-4-((3*R*,7*S*,10*S*,13*R*,17*R*)-7-hydroxy-10,13-dimethyl-3-(4-((((3*R*,6*R*,8a*S*,9*R*,10*S*,12*R*,12a*R*)-3,6,9-trimethyldecahydro-12*H*-3,12-epoxy [1,2]dioxepino [4,3-i]isochromen-10-yl)oxy)methyl)-1*H*-1,2,3-triazol-1-yl)hexadecahydro-1*H*-cyclopenta[a]phenanthren-17-yl)pentanoate (UDCMe-(1,4)-t-DHA)

Flash chromatography (EtOAc/cyclohexane 1:1) afforded 1.28 g of pure compound as a white powder in a 72% yield. The analytical and spectroscopic data were in agreement with the literature [41] (see Figures S1–S3 in Supplementary Materials).

#### 2.1.4. Characterization of Methyl (4*R*)-4-((3*R*,7*R*,10*S*,13*R*,17*R*)-7-hydroxy-10,13-dimethyl-3-(4-((((3*R*,6*R*,8a*S*,9*R*,10*S*,12*R*,12a*R*)-3,6,9-trimethyldecahydro-12*H*-3,12-epoxy [1,2]dioxepino [4,3-i]isochromen-10-yl)oxy)methyl)-1*H*-1,2,3-triazol-1-yl)hexadecahydro-1*H*-cyclopenta[a]phenanthren-17-yl)pentanoate (CDCMe-(1,4)-t-DHA)

Flash chromatography (EtOAc/cyclohexane 1:1) afforded 0.94 g of pure compound as a white powder in a 52% yield.

^1^H NMR (400 MHz, CDCl_3_): δ 7.54 (s, 1H), 5.44 (s, 1H), 4.90 (dd, *J* = 8.0, 4.4 Hz, 2H), 4.64 (d, *J* = 12.4 Hz, 1H), 4.35 (td, *J* = 10.5, 5.5 Hz, 1H), 3.88 (s, 1H), 3.66 (s, 3H), 2.74 (q, *J* = 13.1 Hz, 1H), 2.68–2.59 (m, 1H), 2.43–1.05 (m, 40H), 0.99 (s, 3H), 0.94 (d, *J* = 1.4 Hz, 3H), 0.93 (d, *J* = 1.6 Hz, 3H), 0.87 (d, *J* = 7.3 Hz, 3H), 0.68 (s, 3H). ^13^C NMR (101 MHz, CDCl_3_): δ 174.9, 144.2, 120.5, 104.2, 101.5, 88.1, 81.3, 68.5, 61.6, 61.2, 56.0, 52.7, 51.7, 50.5, 44.6, 42.9, 42.3, 39.6, 39.5, 37.5, 37.0, 36.6, 36.0, 35.5, 35.4, 34.7, 34.4, 33.1, 31.1, 31.1, 31.0, 28.4, 28.3, 26.3, 24.8, 24.6, 23.8, 23.0, 20.8, 20.5, 18.4, 13.1, 11.9. MS (ESI+): *m*/*z* calcd for C_43_H_67_N_3_O_8_ [M + H]^+^: 754.50, found 754.52. (See Figures S4–S6 in Supplementary Materials).

#### 2.1.5. General Procedure for Ruthenium-Catalyzed Azide–Alkyne Cycloaddition (RuAAC) Reactions

DHA-Alk [38] (1.20 mmol) was dissolved in 8 mL of anhydrous THF. To this solution, Cp*RuCl(PPh_3_)_2_ (0.06 mmol) and 1.16 mmol of N_3_-UDCMe [41] or N_3_-CDCMe [42] in anhydrous THF (5 mL) were added. The reaction mixture was stirred at room temperature for 18 h, after which the solvent was removed under vacuum. The crude residue was purified by flash chromatography.

#### 2.1.6. Characterization of Methyl (4*R*)-4-((3*R*,7*S*,10*S*,13*R*,17*R*)-7-hydroxy-10,13-dimethyl-3-(5-((((3*R*,6*R*,8a*S*,9*R*,10*S*,12*R*,12a*R*)-3,6,9-trimethyldecahydro-12*H*-3,12-epoxy [1,2]dioxepino [4,3-i]isochromen-10-yl)oxy)methyl)-1*H*-1,2,3-triazol-1-yl)hexadecahydro-1*H*-cyclopenta[a]phenanthren-17-yl)pentanoate (UDCMe-(1,5)-t-DHA)

Flash chromatography (EtOAc/cyclohexane 1:1) afforded 218.5 mg of pure compound as a white powder in a 25% yield.

^1^H NMR (400 MHz, CDCl_3_): δ 7.61 (s, 1H), 5.35 (s, 1H), 4.95–4.85 (m, 2H), 4.52 (d, *J* = 12.8 Hz, 1H), 4.22 (tt, *J* = 12.0, 4.1 Hz, 1H), 3.66 (s, 3H), 3.65–3.57 (m, 1H), 2.74–2.64 (m, 1H), 2.45–1.05 (m, 40H), 1.03 (s, 3H), 0.93 (dd, *J* = 6.1, 2.0 Hz, 6H), 0.88 (d, *J* = 7.3 Hz, 3H), 0.69 (s, 3H). ^13^C NMR (101 MHz, CDCl_3_): δ 174.8, 133.7, 132.5, 104.5, 101.8, 88.2, 81.0, 71.2, 59.0, 58.0, 55.4, 55.0, 52.5, 51.6, 44.2, 43.9, 43.7, 39.9, 39.2, 37.6, 36.6, 36.4, 36.1, 35.4, 35.2, 34.5, 34.5, 31.2, 31.1, 30.7, 28.7, 27.7, 27.0, 26.2, 24.8, 24.6, 23.7, 21.4, 20.4, 18.5, 13.0, 12.3. MS (ESI+): *m*/*z* calcd for C_43_H_67_N_3_O_8_ [M + H]^+^: 754.50, found 754.53. Elemental analysis calcd. (%) for C_43_H_67_N_3_O_8_: C, 68.50; H, 8.96; N, 5.57; found: C 68.45, H 8.91, N 5.53 (see Figures S7–S9 in Supplementary Materials).

#### 2.1.7. Characterization of Methyl (4*R*)-4-((3*R*,7*R*,10*S*,13*R*,17*R*)-7-hydroxy-10,13-dimethyl-3-(5-((((3*R*,6*R*,8a*S*,9*R*,10*S*,12*R*,12a*R*)-3,6,9-trimethyldecahydro-12*H*-3,12-epoxy [1,2]dioxepino [4,3-i]isochromen-10-yl)oxy)methyl)-1*H*-1,2,3-triazol-1-yl)hexadecahydro-1*H*-cyclopenta[a]phenanthren-17-yl)pentanoate (CDCMe-(1,5)-t-DHA)

Flash chromatography (EtOAc/cyclohexane 1:1) afforded 241.2 mg of pure compound as a white powder in a 28% yield.

^1^H NMR (400 MHz, CDCl_3_): δ 7.60 (s, 1H), 5.37 (s, 1H), 4.96–4.86 (m, 2H), 4.53 (d, *J* = 12.8 Hz, 1H), 4.15 (t, *J* = 12.2 Hz, 1H), 3.85 (s, 1H), 3.67 (s, 3H), 2.97 (q, *J* = 13.1 Hz, 1H), 2.69 (dt, *J* = 8.0, 4.1 Hz, 1H), 2.44–1.06 (m, 40H), 0.99 (s, 3H), 0.95 (s, 3H), 0.93 (s, 3H), 0.89 (d, *J* = 7.3 Hz, 3H), 0.68 (s, 3H). ^13^C NMR (101 MHz, CDCl_3_): δ 174.9, 133.7, 132.5, 104.5, 101.8, 88.2, 81.1, 68.4, 59.8, 58.3, 55.9, 52.6, 51.6, 50.3, 44.3, 42.9, 42.7, 39.7, 39.5, 37.6, 37.4, 36.5, 35.5, 35.5, 34.6, 34.2, 32.9, 31.2, 31.1, 30.8, 28.3, 27.6, 26.3, 24.8, 24.6, 23.8, 23.1, 20.8, 20.4, 18.4, 13.0, 12.0. MS (ESI+): *m*/*z* calcd for C_43_H_67_N_3_O_8_ [M + H]^+^: 754.50, found 754.45. Elemental analysis calcd. (%) for C_43_H_67_N_3_O_8_: C, 68.50; H, 8.96; N, 5.57; found: C 68.42, H 8.91, N 5.50 (see Figures S10–S12 in Supplementary Materials).

### 2.2. Chemical Stability of BA-DHA Hybrids

HPLC-MS/MS analyses were performed on a HPLC Agilent 1260 (Agilent Technologies, Santa Clara, CA, USA) equipped with a diode array UV detector and a mass spectrometer TSQ Quantum Access Max with an electrospray ionization source (Thermo Fisher Scientific, Waltham, MA, USA); 0.5 mL samples were used as sources for the automated injection. LC-MS-grade methanol (MeOH) was purchased from Sigma-Aldrich at the highest available purity and was used without any further purification. Ultrapure water (uH_2_O) was produced using a Sartorius Arium Pro^®^ system. Other solvents and reagents for analytical studies—formic acid (HCOOH), ammonium acetate (NH_4_OAc), and dimethyl sulfoxide (DMSO)—were of HPLC-grade or higher. The chromatographic separation was performed on a reverse-phase Zorbax Eclipse XDB-C8 column 4.6 × 150 mm, 5 mm (Agilent Technologies) at a flow rate of 0.5 mL/min under isocratic conditions: mobile phase A (HCOOH 0.1% and HN_4_OAc 5 mM in uH_2_O *v*/*v*) and mobile phase B (MeOH) 10:90. MS/MS (ESI+) parameters: CDC-DHA precursor ion 681 [M + 23], product ion 260, ce 32 eV; CDCMe-s-DHA precursor ion 795 [M + 23], product ion 411, ce 34 eV; CDCMe-(1,4)-t-DHA precursor ion 776 [M + 23], product ion 261, ce 31 eV; UDCMe-(1,5)-t-DHA precursor ion 776 [M + 23], product ion 261, ce 31 eV; CDCMe-(1,5)-t-DHA precursor ion 754 [M + 1], product ion 163, ce 38 eV.

The quantification of each hybrid in stability studies was carried out using the HPLC-MS/MS method described above.

A DMSO mother solution of the proper hybrid at 20 mM concentration was prepared. Each solution was diluted in complete cell culture medium in Dulbecco’s Modified Eagle Medium (DMEM-HG, Gibco^TM^, Thermo Fisher Scientific Inc.) supplemented with 10% depleted Fetal Bovine Serum (FBS-C) (South America, Thermo Fisher Scientific Inc.) and GlutaMax (Thermo Fisher Scientific Inc.) to a final concentration of 20 μM. A time course at 0, 2, 15, and 24 h was considered. The concentration of the proper hybrid over time was calculated by comparison with that of the initial solution (time 0). The experiment was performed in triplicate (RSD < 10%).

### 2.3. Biological Evaluation

#### 2.3.1. Cell Lines

The biological effects of DHA and of all newly synthesized DHA hybrids were evaluated on two in vitro models of human colon carcinoma, the HCT116 and the RKO cell lines, both purchased from the American Type Culture Collection (ATCC, Manassas, VA, USA).

HCT116 and RKO cells were routinely cultured in Dulbecco’s Modified Eagle Medium (DMEM; Corning, Glendale, AZ, USA) supplemented with 10% fetal bovine serum (FBS; Gibco, Grand Island, NY, USA), 2 mM L-glutamine, 100 U/mL penicillin, and 100 mg/mL streptomycin (L-Glutamine–Penicillin–Streptomycin solution from Sigma-Aldrich, St. Louis, MO, USA).

Human Umbilical Vein Endothelial Cells (HUVECs) were purchased from Lonza (Basilea, Switzerland) and grown on fibronectin (Sigma-Aldrich)-coated flasks or well plates (1 µg/cm^2^) using complete EGM-2 medium (Lonza) containing 2% FBS, hydrocortisone, hFGF (Human Fibroblast growth factor), VEGF (Vascular Endothelial Growth Factor), R3-IGF-1 (Recombinant analog of human Insulin-like Growth Factor-I), ascorbic acid, hEGF (Human Epidermal Growth Factor), GA-1000 (Gentamicin and Amphotericin), and heparin. HUVECs were used between passages 1 and 15.

All cell lines were maintained at 37 °C in an atmosphere of 5% CO_2_ and 90% relative humidity.

#### 2.3.2. Cell Treatments

To evaluate the biological effects of the hybrids and to compare them with DHA antitumor activity, all the molecules were reconstituted in DMSO, whose final concentration during the cell treatments never exceeded 0.1% of the medium volume.

In the first set of experiments, HCT116 and RKO cells were treated with 50 μM DHA or 5 μM of DHA hybrids for 24 or 36, or 72 h, depending on the type of analysis that was performed. Indeed, afterward, the drug’s effects on cell viability, proliferation, cell cycle, or migration/invasion capability were tested. The DHA and hybrids’ concentrations used for the experiments and the analysis timepoints were determined in preliminary dose–effect and time-course tests performed on both cell lines. In all experiments, the biological effects of the ursodeoxycholic and chenodeoxycholic bile acids (UDCA and CDCA, respectively), used at the same concentration of hybrids (5 μM) were evaluated, whereas DMSO was used as a control (CTRL).

For IC_50_ determination, HCT116 and RKO cell lines were treated with a scalar dose of DHA and hybrids for 72 h, and then cell proliferation was analyzed by MTT test.

For the experiments on HUVECs, cells were treated with the same concentration of DHA and selected hybrids used for HCT116 and RKO cell lines for 72 h to evaluate their effects on cell proliferation, metabolic activity, and expression of VEGF receptor-2 (VEGFR-2).

#### 2.3.3. Analysis of Cell Viability, Proliferation, Cell Cycle, and Apoptosis Induction

After 36 h of treatment, flow cytometry was used to analyze the effects of DHA and hybrids on HCT116 and RKO cell cycles. For this purpose, as previously described [43], cells were incubated for 1.5 h at 37 °C with 5-bromodeoxyuridine (BrdU), which was incorporated into newly synthesized DNA during replication. BrdU incorporation was detected using a primary anti-BrdU antibody (BioLegend, San Diego, CA, USA) followed by an FITC-conjugated secondary antibody (Tonbo Biosciences, San Diego, CA, USA) for visualization. Subsequently, cells were stained with propidium iodide (PI, 50 μg/mL, Sigma-Aldrich) and analyzed using the FACSCalibur flow cytometer and the CellQuest software, version 6.1 (BD Biosciences, San Josè, CA, USA) to determine the distribution of cells in the different cell cycle phases. Data analysis was carried out using the FlowJo™ v10.10 Software (BD Life Sciences, Franklin Lakes, NJ, USA).

After 72 h of treatment, instead, cell viability and proliferation were evaluated on HCT116, RKO, and HUVEC cell lines by Trypan blue dye exclusion count and MTT colorimetric assay (Roche Diagnostics Corporation, Indianapolis, IN, USA), observing the manufacturer’s instructions. MTT assay quantification was performed using a TECAN Infinite^®^ M Plex microplate reader (Tecan Trading AG, Männedorf, Switzerland).

To confirm DHA and hybrid effects on HUVEC, cell morphology was observed, and phase-contrast images were acquired using an EVOS XL microscope system (Advanced Microscopy Group, AMG, Bothell, WA, USA).

At the same timepoint, the potential pro-apoptotic effect of DHA hybrids was assessed by flow cytometry after the double-staining of HCT116 and RKO cells using an annexin V-FITC/propidium iodide (PI) kit (Beckman Coulter Inc., Brea, CA, USA), following the manufacturer’s instructions. Also, in this case, data were obtained by using a FACSCalibur flow cytometer (BD Biosciences) and analyzed with the FlowJo™ Software (BD Life Sciences).

#### 2.3.4. Real-Time Analysis of the Influence of DHA Hybrids on HCT116 Cell Migration and Invasion

HCT116 real-time migration was assessed using an xCELLigence RTCA DP Instrument (Agilent), using the specific CIM-Plates (Agilent), electronically integrated Boyden Chambers. In particular, in these assays, the xCELLigence RTCA DP Instrument registers, in real time, impedance values that are related to cell migration, and then converts them into a dimensionless parameter, that is, the “Cell Index” (CI).

For migration experiments, HCT116 cells were treated for 24 h in 6 wells in complete DMEM medium with DHA, DHA hybrids, UDCA, CDCA, and the vehicle as described above. After treatments, cells were maintained in DMEM with 1% FBS and antibiotics for 2 h before being detached and accurately counted. Therefore, an equal number of cells (3 × 10^4^ cells/well) was seeded in the upper chamber of the CIM-Plates, using DMEM containing 1% FBS and antibiotics, following the manufacturer’s instructions. The lower chambers of the CIM-Plates were filled with DMEM added with 10% FBS and antibiotics. Finally, plates were inserted into the instruments at 37 °C in the presence of 5% CO_2_ and 90% of relative humidity, and migration data were recorded every 5 min.

The xCELLigence RTCA DP Instrument (Agilent) and CIM-plates were also employed for invasion assays, following the same experimental protocol used for migration evaluation, but in this case, the bottom of the upper chambers was covered with a layer of 1:20 diluted Matrigel (BD Biosciences). Also, for invasion assays, data were recorded every 5 min.

#### 2.3.5. Real-Time Analysis of the Influence of DHA Hybrids on HUVEC Proliferation

The time-course effects of DHA and hybrids on HUVEC proliferation were evaluated using the xCELLigence RTCA DP Instrument (Agilent) and specific E-Plates (Agilent). First, we added 50 μL of EGM-2 complete medium to the E-Plates wells, and then they were connected to the xCELLigence system to record the background measurements of the wells before adding HUVECs (4 × 10^3^ cells/well) resuspended in 100 μL of complete medium. Cells were allowed to settle for 30 min at room temperature before being added to each well of the E-Plates with 50 μL of medium containing treatments and then inserting the plates in the instrument at 37 °C in the presence of 5% CO_2_ and 90% relative humidity for the real-time analysis until 72 h. Specifically, cells were treated with DHA, selected DHA hybrids, UDCA, CDCA, and the vehicle as described above. For the cell proliferation assays, the xCELLigence RTCA DP Instrument registers the impedance values related to cell viability and proliferation in real time, converting them into Cell Index (CI). In these experiments, impedance was measured every 5 min.

#### 2.3.6. Cytometric Analysis of HUVEC VEGF Receptor-2 Expression

The potential modulation of DHA and hybrids on VEGF receptor-2 (VEGFR-2) expression in HUVECs was evaluated by flow cytometry. For this purpose, cells were treated for 72 h with DHA, selected hybrids, UDCA, CDCA, and the vehicle. Cells were then harvested upon trypsinization, washed with PBS (Phosphate-Buffered Saline, Euroclone, Milan, Italy), and centrifuged at 300× *g* for 5 min. Next, cells were stained as described below. Flow cytometric immunophenotyping was performed according to standard protocols with pre-titered PE Mouse Anti-Human CD309 (VEGFR-2, clone ES8-20E6) (Miltenyi Biotec, Bergisch Gladbach, Germany). To exclude dead cells, the fixable viability dye LIVE/DEAD™ Fixable Far Red Dead Cell Stain Kit (Thermo Fisher Scientific) was added to the staining mix and incubated at RT for 15 min, then washed again and acquired immediately on a BD FACSCalibur flow cytometer using the CellQuest software (all from BD Biosciences). Data analysis was performed using the FlowJo™ Software (BD Life Sciences). VEGFR-2 expression level was quantified by statistically assessing the difference in mean fluorescence intensities between stained and unstained populations using a modified resolution metric equation from Ortyn et al. [44]. The modification involved adjusting standard deviations for population size, as expressed by the following test for the difference between the means of samples with unknown variances: expression level = (mean stained − mean unstained)/√((SD^2^ stained/N stained) + (SD^2^ unstained/N unstained).

#### 2.3.7. Statistical Analysis

For the evaluation of the hybrids’ biological effects, data obtained from at least three independent experiments were analyzed (unless otherwise specified) by one-way ANOVA, followed by the Bonferroni post hoc test (for multiple corrections) using GraphPad Prism software, version 8.4.2 (GraphPad Software, San Diego, CA, USA). Results were expressed as mean ± standard deviation (SD) of replicate experiments. Statistical significance was defined as almost *p* < 0.05.

IC_50_ values were calculated using GraphPad Prism software, version 8.4.2 (GraphPad Software).

## 3. Results

### 3.1. Synthesis of BA-DHA Hybrids

**Scheme 1:**
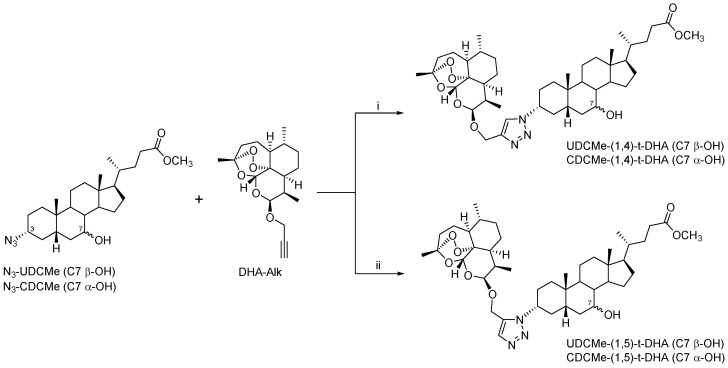
Synthesis of triazolyl hybrids. (i) Selected azide (1 eq), DHA-Alk (1.1 eq), CuSO_4_*5H_2_O (0.05 eq), sodium ascorbate (0.15 eq), CH_2_Cl_2_/CH_3_CN/H_2_O (1:1:0.1), 1 h (52–72% yield); (ii) Cp*RuCl(PPh_3_)_2_ (0.05 eq), DHA-Alk (1.05 eq), selected azide (1 eq), THF, 18 h (25–28% yield). The synthesis of UDCMe-(1,4)-t-DHA was previously reported [41].

The triazole-linked hybrids UDCMe-(1,5)-t-DHA, CDCMe-(1,4)-t-DHA, and CDCMe-(1,5)-t-DHA, newly reported in the present work, were prepared via click chemistry, as depicted in Scheme 1. In particular, click hybrids UDCMe-(1,5)-t-DHA and CDCMe-(1,5)-t-DHA were synthesized by following a typical RuAAC procedure, which involved the reaction of the DHA-Alk alkyne with the appropriate N_3_-BA azide in the presence of catalytic amounts of Cp*RuCl(PPh_3_)_2_ in a non-protic solvent as tetrahydrofuran (THF), at room temperature (Scheme 1). The reaction afforded the desired compounds with moderate yields (25–28%) after purification by flash chromatography. Nevertheless, attempts to achieve a higher conversion rate through microwave or conventional heating were unsuccessful due to the decomposition of DHA-Alk under the reaction conditions. The hybrid CDCMe-(1,4)-t-DHA was obtained using the same CuAAC procedure previously reported by our laboratory for the synthesis of the analog UDCMe-(1,4)-t-DHA [41], employing the corresponding N_3_-CDCMe azide as the coupling partner (Scheme 1).

The previously reported hybrids UDC-DHA, CDC-DHA, UDCMe-s-DHA, CDCMe-s-DHA, and UDCMe-(1,4)-t-DHA were synthesized according to procedures described by our group [38,41].

### 3.2. Chemical Stability in Cell Culture Medium

**Figure 2:**
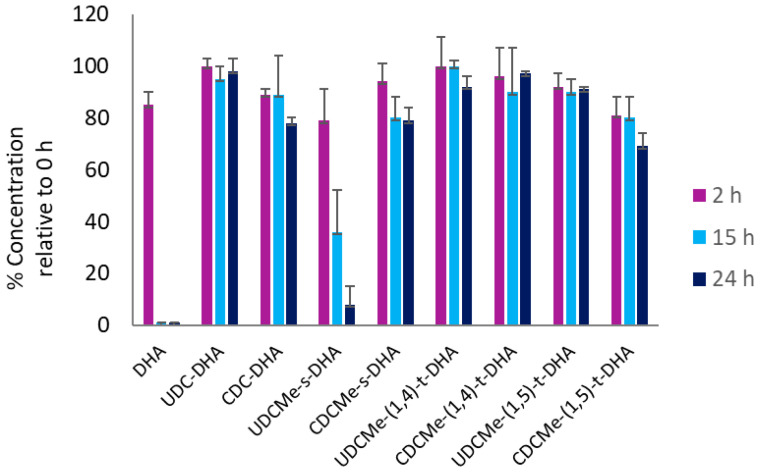
Stability of DHA and BA-DHA hybrids in cell culture medium assessed by HPLC-MS/MS analyses. Stability of DHA and hybrid DHA-UDC was reported in [40]; stability of hybrid UDCMe-(1,4)-t-DHA was reported in [39]; stability of hybrid UDCMe-s-DHA was reported in [41]; stability of hybrids CDC-DHA, CDCMe-s-DHA, CDCMe-(1,4)-t-DHA, UDCMe-(1,5)-t-DHA, and CDCMe-(1,5)-t-DHA was recorded at 0, 2, 15, and 24 h. The data are presented as mean ± SD of three independent experiments.

The chemical stability of UDC-DHA, UDCMe-s-DHA, and UDCMe-(1,4)-t-DHA, as well as unconjugated DHA, in cell culture medium at 37 °C, was evaluated by HPLC–MS/MS analyses in our previous studies [39,40,41]. Following the same protocol, we investigated the chemical stability of CDC-DHA and CDCMe-s-DHA, as well as the newly synthesized CDCMe-(1,4)-t-DHA, UDCMe-(1,5)-t-DHA, and CDCMe-(1,5)-t-DHA, in cell culture medium at 37 °C using HPLC–MS/MS analyses. A time-course study performed at 0, 2, 15, and 24 h revealed that the hybrids CDC-DHA, CDCMe-s-DHA, and CDCMe-(1,5)-t-DHA underwent 22%, 21%, and 31% degradation, respectively, after 24 h of incubation (Figure 2). Hybrids UDC-DHA and UDCMe-(1,4)-t-DHA, previously studied under comparable conditions, were found to be stable for up to 24 h incubation in cell culture medium [39,40], whereas UDCMe-s-DHA underwent decomposition, showing a half-life time of ca. 15 h [41]. Notably, parent DHA was found far more unstable, being completely decomposed after 15 h incubation time (Figure 2) [40]. The overall data indicate that MH actually improved the chemical stability with respect to parent DHA, although to different extents depending on the bile acid moiety and linker type. In particular, UDCMe-s-DHA was found to be the most unstable hybrid, undergoing 82% degradation after 24 h, whereas the corresponding CDC-based analog, CDCMe-s-DHA, exhibited a lower degree of instability, with 31% degradation after 24 h. In contrast, the UDC-based hybrids, UDCMe-(1,5)-t-DHA and the fused UDC-DHA, remained chemically stable after 24 h of incubation, whereas their corresponding CDC analogs, CDCMe-(1,5)-t-DHA and CDC-DHA, exhibited 31% and 21% degradation, respectively, under the same conditions. Notably, both (1,4)-t-click hybrids, UDCMe-(1,4)-t-DHA and CDCMe-(1,4)-t-DHA, demonstrated complete stability after 24 h of incubation.

### 3.3. In Vitro Evaluation of BA-DHA Hybrids’ Effects 

#### 3.3.1. Evaluation of the Antiproliferative Activity of BA-DHA Hybrids in HCT116 and RKO Cells

**Figure 3:**
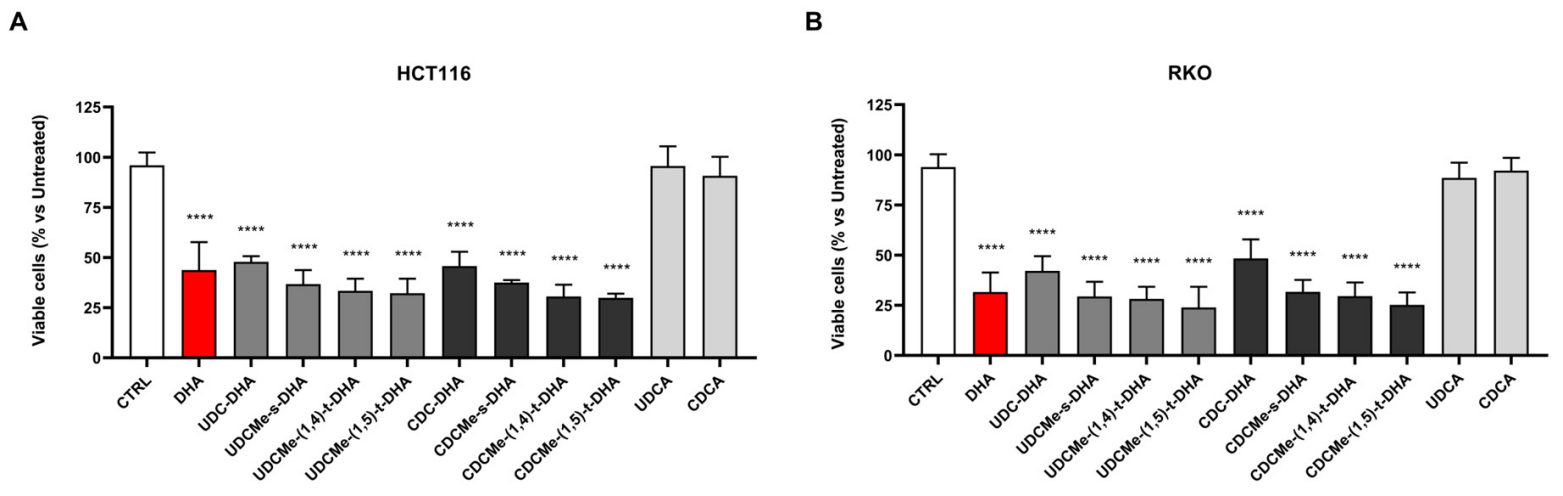
Effects of DHA and DHA hybrids on HCT116 and RKO viable cells’ number. Cell viability was evaluated in HCT116 cells (**A**) and RKO cells (**B**) treated for 72 h with 50 μM DHA and 5 μM BA-DHA hybrids or BAs alone. Results are reported as mean ± SD of at least three independent experiments. Statistical analysis was performed by one-way ANOVA followed by the Bonferroni post hoc test. **** *p* < 0.0001 with respect to control (CTRL).

In an initial set of experiments aimed at verifying the biological properties of the BA-DHA hybrids, we analyzed their effects on cell viability and metabolic activity, cell cycle phases’ distribution, and apoptosis induction in two colon carcinoma cell lines, HCT116 and RKO. The concentrations used in all experiments were set at 5 μM for the BA-DHA hybrids as well as for BAs CDCA and UDCA, and 50 μM for unconjugated DHA, based on preliminary experiments and according to the protocols reported in our previous BA-DHA hybrid studies [38,39,40]. Observing HCT116 cells after 72 h of treatment, we found that all BA-DHA hybrids at 5 μM showed a potent and significant cytostatic effect, reducing the number of viable cells in a comparable or superior manner with respect to DHA at 50 μM (Figure 3A). Among all hybrids, the click compounds UDCMe-(1,4)-t-DHA, CDCMe-(1,4)-t-DHA, UDCMe-(1,5)-t-DHA, and CDCMe-(1,5)-t-DHA exhibited a slightly higher effect in reducing viable cells, also compared to unconjugated DHA (Figure 3A).

On the other hand, treatment with DHA at 5 μM resulted in a markedly lower reduction in viable cell number (Figure S13A), further confirming the superior efficacy of the hybrid compounds. Analysis of HCT116 metabolic activity, assessed by the MTT assay, confirmed that the significant effect of the BA-DHA hybrids at 5 μM was comparable to that of DHA at 50 μM after 72 h of treatment (Figure S14A). This equivalence was further supported by statistical analysis of the MTT results obtained with DHA and the hybrids (Table S1).

Regarding the RKO cell line, viability data confirmed the strong and significant cytostatic effects of the hybrids after 72 h of treatment, with all BA-DHA hybrids being more effective than DHA. In particular, the click hybrids UDCMe-(1,5)-t-DHA and CDCMe-(1,5)-t-DHA exhibited the highest effect in reducing the number of viable cells (Figure 3B). The same trend was also clearly shown by MTT results, obtained at the same timepoint (Figure S14B), and by their statistical analysis (Table S1). Also in this case, the analysis of the effect of DHA 5 μM on viable cells’ number (Figure S13B) emphasized the greater effectiveness of the hybrids with respect to DHA alone.

Analysis of IC_50_ values highlighted and underlined that all hybrids were notably more effective than DHA (Table 1).

In both cell lines UDCA and CDCA alone, when used at 5 µM, this did not significantly impair either viable cell number or metabolic activity when compared to the control (Figure 3 and Figure S14).

#### 3.3.2. Cytostatic and Cytotoxic Activity of BA-DHA Hybrids

**Figure 4:**
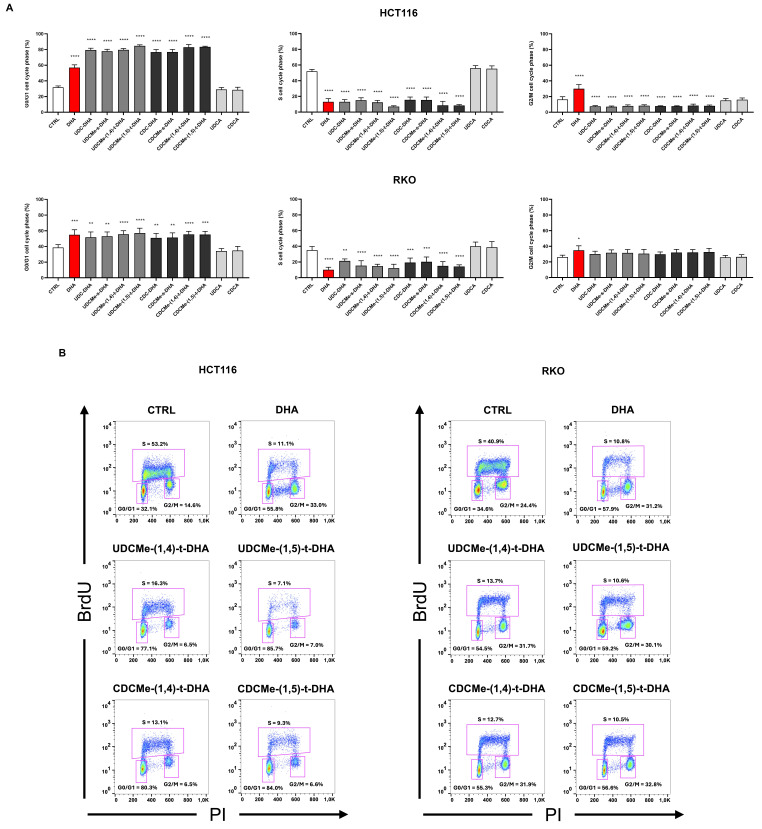
Effects of DHA and BA-DHA hybrids on HCT116 and on RKO cell cycle. (**A**) Cells’ distribution in the cell cycle phases after treatment for 36 h with 50 μM DHA or 5 μM BA-DHA hybrids and BAs alone; (**B**) a representative cytometric panel of HCT116 and RKO cell cycle after treatment for 36 h with DHA (50 μM) or selected BA-DHA hybrids (5 μM). In (**A**), results are reported as mean ± SD of at least three independent experiments. Statistical analysis was performed by one-way ANOVA followed by the Bonferroni post hoc test. * *p* < 0.05, ** *p* < 0.01, *** *p* < 0.001, **** *p* < 0.0001 with respect to control (CTRL).

The potential effect of BA-DHA hybrids on cell cycle 36 h post-treatment was also studied. The results revealed that in both cell lines, all hybrids could cause a significant arrest of the cell cycle in the G0/G1 phase with respect to the control (CTRL), deeply reducing the S phase, justifying the strong viable cell number reduction observed at 72 h of treatment (Figure 4A,B and Figure S15). Moreover, also in the case of cell cycle analysis, the cytostatic effect of BA-DHA hybrids at 5 μM was comparable to that of DHA used at 50 μM, while the unconjugated BAs, UDCA and CDCA did not exert any significant effect as described above (Figure 4A,B and Figure S15).

**Figure 5:**
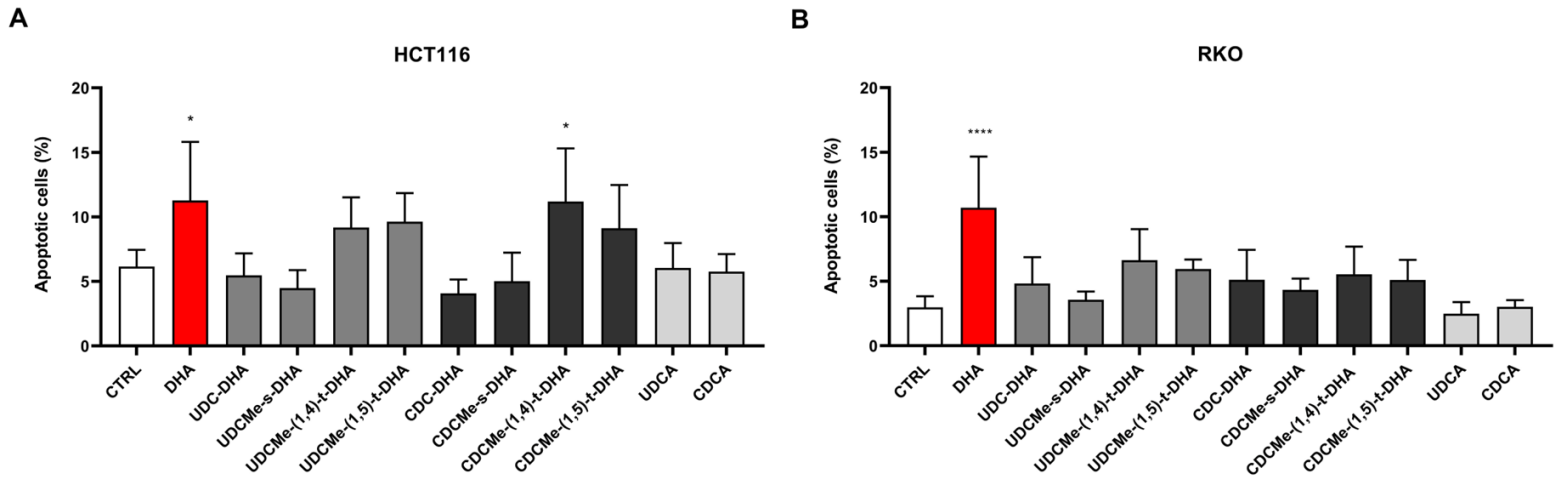
(**A**) Effects of DHA and BA-DHA hybrids on apoptosis induction in HCT116; (**B**) effects of DHA and BA-DHA hybrids on apoptosis induction in RKO cell lines. Apoptosis induction in HCT116 and RKO cell lines treated for 72 h with 50 μM DHA or 5 μM BA-DHA hybrids and BAs alone was evaluated. Results are reported as mean ± SD of at least three independent experiments. Statistical analysis was performed by one-way ANOVA followed by the Bonferroni post hoc test. * *p* < 0.05, **** *p* < 0.0001 with respect to control (CTRL).

Evaluation of apoptosis induction revealed that the click hybrids UDCMe-(1,4)-t-DHA, UDCMe-(1,5)-t-DHA, CDCMe-(1,4)-t-DHA, and CDCMe-(1,5)-t-DHA at 5 μM promoted apoptosis in HCT116 cells to an extent comparable to unconjugated DHA at 50 μM, whereas fused hybrids UDC-DHA and CDC-DHA, as well as succinyl-linked hybrids UDCMe-s-DHA and CDCMe-s-DHA, did not exhibit a pro-apoptotic effect (Figure 5A). In contrast, in RKO cells, all tested hybrids induced apoptosis to a lesser extent than DHA (Figure 5B).

#### 3.3.3. BA-DHA Hybrids’ Effects on Cell Migration and Invasion

**Figure 6:**
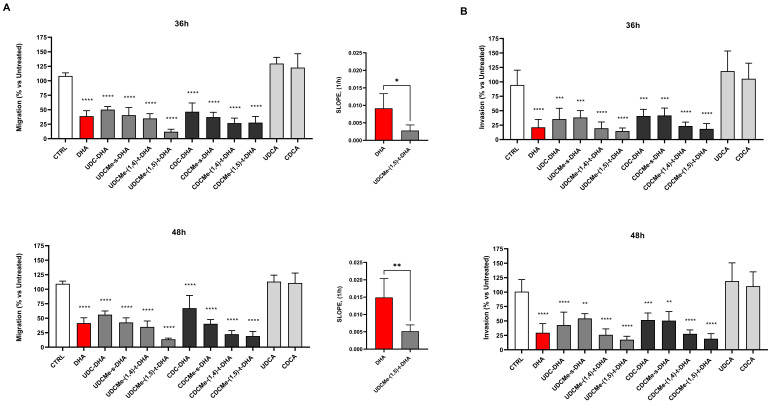
Evaluation of the effects of DHA and BA-DHA hybrids on HCT116 migration and invasion capability. Cell migration (**A**) and invasion (**B**) were evaluated in real time by a xCELLigence RTCA DP Instrument. Histograms show the analysis of the effect of 24 h pretreatments with DHA and BA-DHA hybrids or BAs alone at the concentrations of 50 μM and 5 μM, respectively, after 36 and 48 h of cell migration (**A**) and invasion (**B**). In (**A**), slope values, describing the steepness, incline, gradient, and changing rate of the CI curves over time, are reported for migration of cells pretreated with DHA and UDCMe-(1,5)-t-DHA. Results are reported as mean ± SD of at least three independent experiments. Statistical analysis was performed using one-way ANOVA followed by the Bonferroni post hoc test or Student’s *t*-test (for slope values analysis). * *p* < 0.05, ** *p* < 0.01, *** *p* < 0.001, **** *p* < 0.0001 with respect to the control (CTRL).

The potential effect of BA-DHA hybrids on HCT116 cell migration was analyzed in real time. The BA-DHA hybrids, tested at 5 μM, as well as DHA, tested at 50 μM, demonstrated the ability to markedly decrease the cell migration rate. In particular, UDCMe-(1,5)-t-DHA resulted in the most effective hybrid (Figure 6A) and showed a significantly higher migration-inhibitory activity with respect to DHA, as shown also by slope values (Figure 6A). On the other hand, no significant effect was exerted by both unconjugated UDCA and CDCA during the entire time course of the experiments.

Invasion assays showed similar results: indeed, all BA-DHA hybrids considerably inhibited HCT116 invasion capability, and UDCMe-(1,5)-t-DHA seemed to be the most effective (Figure 6B).

#### 3.3.4. Evaluation of the Click Hybrids’ Inhibition of Endothelial Cell Proliferation and Down-Modulation of the Expression of VEGF Receptor-2

For further mechanistic investigations, the click BA-DHA hybrids UDCMe-(1,4)-t-DHA, CDCMe-(1,4)-t-DHA, UDCMe-(1,5)-t-DHA, and CDCMe-(1,5)-t-DHA were selected based on their favorable stability and biological activity profiles, including their effects on tumor cells’ proliferation and migration/invasion. In particular, we investigated the potential activity of these selected hybrids on the vascular compartment, which has a fundamental role in tumor progression, testing their effects, first of all, on endothelial cells’ proliferation and metabolic activity.

**Figure 7:**
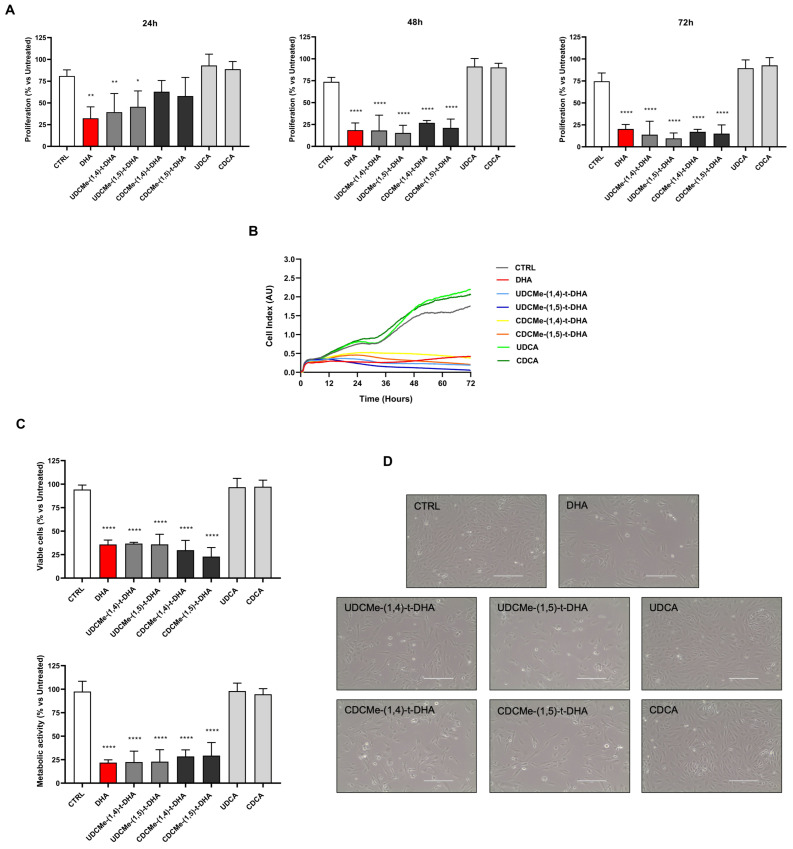
Evaluation of the effects of DHA and BA-DHA hybrids on HUVECs. (**A**) Cell proliferation evaluated in real time by xCELLigence RTCA DP Instrument. Histograms reported the analysis of the CI values at 24, 48, and 72 h after treatment with 50 μM DHA or 5 μM selected BA-DHA hybrids or BAs alone. (**B**) Representative xCELLigence RTCA DP Instrument time-course of HUVEC proliferation. (**C**) HUVECs’ viability and metabolic activity were analyzed after 72 h of the indicated treatments. In (**A**,**C**), results are reported as mean ± SD of at least three independent experiments. Statistical analysis was performed by ANOVA followed by Bonferroni post hoc test. * *p* < 0.05, ** *p* < 0.01, **** *p* < 0.0001 with respect to control (CTRL). (**D**) Representative panel of phase-contrast images of HUVECs exposed for 72 h to the indicated treatments (bar = 200 μm).

Real-time analysis of HUVEC proliferation evaluated using the xCELLigence RTCA DP Instrument, confirmed that DHA and all tested BA-DHA click hybrids markedly reduced cell proliferation (Figure 7A,B), although it should be still underlined that DHA was tested at a 10-fold higher concentration than the BA-DHA click hybrids. It is worth noting that, at later timepoints, the click hybrids UDCMe-(1,4)-t-DHA, CDCMe-(1,4)-t-DHA, UDCMe-(1,5)-t-DHA, and CDCMe-(1,5)-t-DHA, used at 5 μM, were able to inhibit cell proliferation to an extent comparable to, or even greater than that of DHA used at 50 μM (Figure 7A,B).

Evaluation of the number of viable cells and metabolic activity after 72 h of treatment supported the findings obtained from real-time proliferation assays (Figure 7C), confirming that the treatment of HUVECs with UDCMe-(1,4)-t-DHA, CDCMe-(1,4)-t-DHA, UDCMe-(1,5)-t-DHA, and CDCMe-(1,5)-t-DHA at 5 μM was as effective as DHA administered at a 10-fold higher concentration (Figure 7C). Moreover, CDCMe-(1,5)-t-DHA was found to reduce the number of viable cells more efficiently than the other click hybrids tested, with statistically significant differences compared to UDCMe-(1,4)-t-DHA (*p* = 0.0486; CDCMe-(1,5)-t-DHA vs. UDCMe-(1,4)-t-DHA, *t*-test) and DHA (*p* = 0.0236; CDCMe-(1,5)-t-DHA vs. DHA, *t*-test) (Figure 7C). UDCA and CDCA, instead, as expected, did not affect proliferation nor their viability or metabolic activity. Data concerning the effects of UDCMe-(1,4)-t-DHA, CDCMe-(1,4)-t-DHA, UDCMe-(1,5)-t-DHA, and CDCMe-(1,5)-t-DHA on HUVEC viability and metabolic activity were also supported by morphological analysis of HUVEC, performed at the same timepoint (Figure 7D).

**Figure 8:**
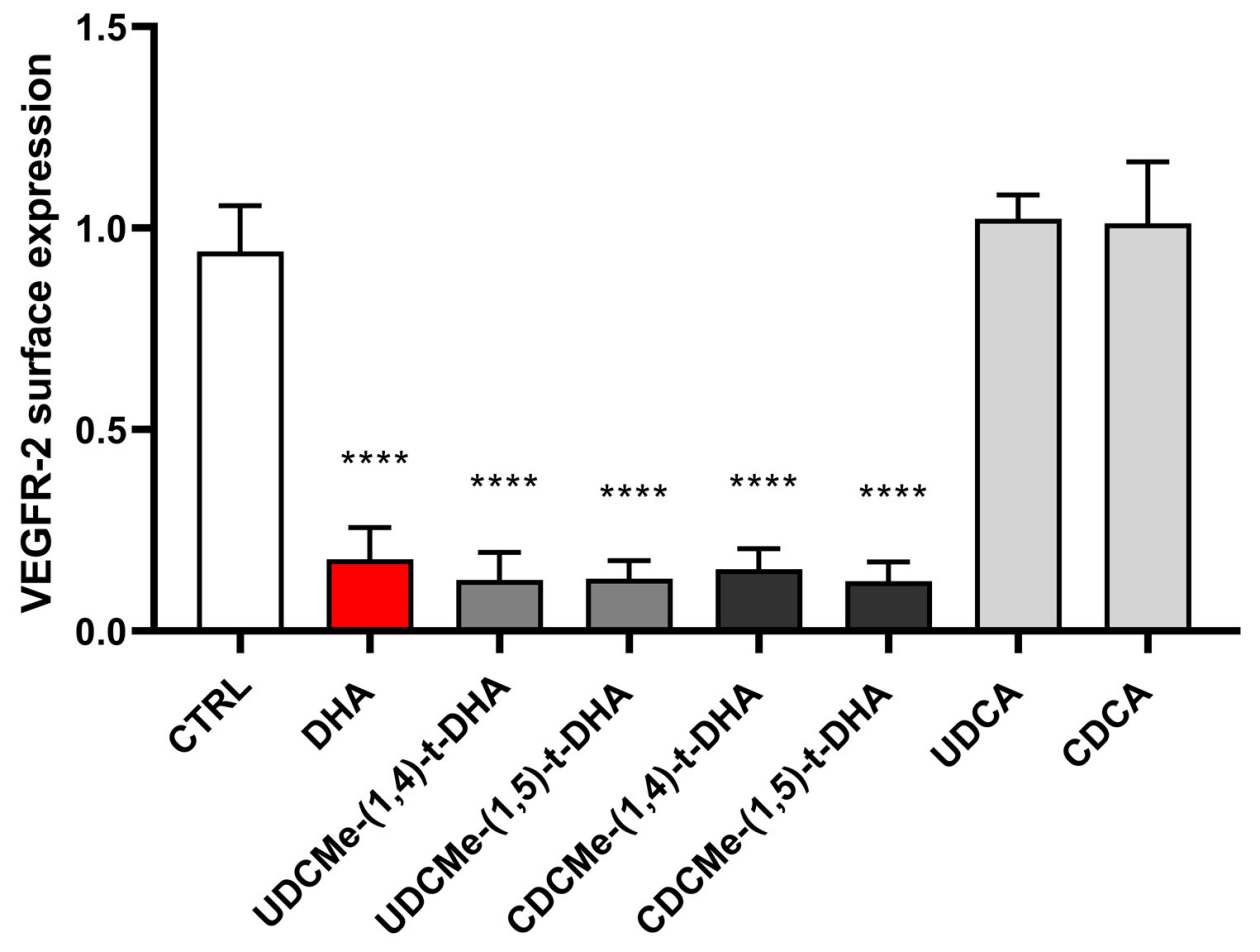
Evaluation of the effects of DHA and BA-DHA hybrids on VEGFR-2 expression in primary HUVECs. VEGFR-2 expression was evaluated by flow cytometry after 72 h of treatment with 50 μM DHA or 5 μM of selected BA-DHA hybrids or BAs alone. Results, expressed as fold of untreated cells’ VEGFR-2 expression, are reported as mean ± SD of at least three independent experiments. Statistical analysis was performed using one-way ANOVA followed by the Bonferroni post hoc test. **** *p* < 0.0001 with respect to the control (CTRL).

In the same set of experiments, we also investigated the possible ability of the click hybrids to modulate the surface expression of VEGF receptor-2 (VEGFR-2). The cytometric analysis of VEGFR-2 revealed that all hybrids tested at 5 μM could strongly down-modulate the surface expression of this receptor, and their effect was similar to VEGFR-2 down-modulation induced by DHA at 50 μM (Figure 8).

## 4. Discussion

Our interest and synthetic expertise in natural endogenous molecules such as BAs prompted us to design NP-NP hybrids through the molecular hybridization of DHA with a variety of BAs that have already shown promising biological activity in leukemia [35,38] and hepatocellular carcinoma [38,39,40] cells. In the present work, UDCA and CDCA were selected for MH with DHA based on their intrinsic properties. Indeed, UDCA and CDCA, although differing only in the absolute configuration at C7 (Figure 1A), exhibit distinct physicochemical properties, with CDCA being more hydrophobic than UDCA [45]. The degree of hydrophobicity has been recognized as a fundamental factor influencing BAs’ different potential anticancer activity [22,30,33]. Nevertheless, the protective or toxic properties of BAs remain controversial, being correlated with many factors, including the concentration and type of BA [33,34,46,47].

Among the endogenous bile acids, UDCA, naturally present in small amounts in normal human bile, has the weakest hydrophobicity. Owing to its favorable safety and tolerability profile, UDCA has been extensively studied in recent years for the treatment of cholestatic liver diseases and has been approved by the FDA for clinical use [48]. One of the most attractive therapeutic potentials of UDCA lies in its antiproliferative and pro-apoptotic effects, as reported in several studies involving different cancer cell types [49,50,51,52,53]. In particular, previous studies have demonstrated its chemopreventive effect in CRC development [51]. On the other hand, CDCA is one of the two main primary bile acids in human and animal bile [54], which has been applied in the clinical treatment of cholesterol gallstones for a long history [55]. CDCA is a hydrophobic bile acid, and its cytotoxic effects have been reported in several human CRC cells via oxidative stress [55,56]. Moreover, CDCA is a high-affinity endogenous FXR (Farnesoid X Receptor) ligand that regulates metabolic homeostasis at physiological concentrations and, through FXR signaling, has been associated with antiproliferative and pro-apoptotic effects in cholangiocarcinoma cells and mouse models [57]. To clarify the effective role of BAs in improving the pharmacological profile of DHA in CRC cells, we designed a selection of BA-DHA hybrids, taking into account not only the nature of the BA but also the conjugation site on the BA scaffold, the presence or absence of a linker, and the type of linker employed (Figure 1B). In particular, fused hybrids UDCMe-DHA and CDCMe-DHA have been obtained by condensation at C24 of the corresponding BA between the two pharmacophore units by means of an enzymatic labile ester bond [38] (Figure 1B); similarly, UDCMe-s-DHA [41] and CDCMe-s-DHA [38] present an enzymatic labile ester bond, but the two pharmacophore units have been conjugated at C3 of the corresponding BA using a succinyl linker (Figure 1B). Among the non-cleavable linker-based hybrids, we investigated two series of DHA conjugates, UDCMe-(1,4)-t-DHA [41] and UDCMe-(1,5)-t-DHA, as well as CDCMe-(1,4)-t-DHA and CDCMe-(1,5)-t-DHA, incorporating both 1,4- and 1,5-disubstituted triazole linkers (Figure 1B). Notably, the hybrids UDCMe-(1,5)-t-DHA, CDCMe-(1,4)-t-DHA, and CDCMe-(1,5)-t-DHA are reported here for the first time (Scheme 1). This design also allows for exploring how the nature of BAs and the structural diversity between the 1,4- and 1,5-isomers of 1,2,3-triazole can influence their biological activity, keeping in mind the properties of triazole scaffolds, which are among the most widely applied bioisostere moieties in medicinal chemistry for identifying new potential drug candidates [58,59,60]. In fact, the structural features of 1,2,3-triazoles, such as rigidity, polarity, and the ability to participate in hydrogen bonding formation, enable them to mimic different functional groups (e.g., esters and amides) with the advantage of marked biological and chemical stability, resulting in more efficient drug candidates. For example, 1,4- and 1,5-disubstituted 1,2,3-triazoles are considered good bioisosteres of *trans*- and *cis*-amide bonds, respectively, with improved metabolic stability [61].

All BA-DHA hybrids (Figure 1B) were evaluated for the first time in two CRC cell lines, HCT116 and RKO. HCT116 cells exhibit more epithelial features, whereas RKO cells display a mesenchymal-like phenotype. Both cell lines have invasive potential; therefore, they also represent a suitable model for investigating cell migration and invasion, which are essential steps in the metastatic spread of cancer cells from the primary tumor into surrounding tissues as well as distant organs [62].

For the first time, we demonstrated that all the BA-DHA hybrids tested were more active than DHA alone, showing a 5- to 20-fold increase in HCT116 cells and a 3- to 6-fold increase in RKO cells (Table 1).

With regard to HCT116 cells, the CDCMe-(1,4)-t-DHA hybrid was found to be the most active with IC_50_ = 0.523 μM corresponding to a 20-fold increase with respect to parent DHA, followed by UDCMe-(1,4)-t-DHA (IC_50_ = 0.692 μM, 15-fold increase), UDCMe-(1,5)-t-DHA (IC_50_ = 0.727 μM, 14-fold increase), and CDCMe-(1,5)-t-DHA (IC_50_ = 0.819 μM, 13-fold increase). Fused hybrid UDC-DHA showed comparable cytotoxicity, displaying IC_50_ = 0.876 μM corresponding to a 12-fold increase, whereas CDC-DHA showed a lower effectiveness with IC_50_ = 1.332 μM corresponding to an 8-fold increase. The succinyl linker hybrids UDCMe-s-DHA and CDCMe-s-DHA showed the lowest activity, with a 5-fold increase in their effectiveness with respect to DHA.

In our previous studies, we demonstrated that DHA is chemically and biologically unstable, while UDC-based hybrids such as fused UDC-DHA and the click UDCMe-(1,4)-t-DHA were found to be stable for up to 24 h when incubated in cell culture medium at 37 °C [39,40]. Conversely, UDCMe-s-DHA displayed a half-life of approximately 15 h under similar conditions [41]. To elucidate whether, and to what extent, the chemical stability of these hybrids correlates with their enhanced biological activity compared with unconjugated DHA, we investigated the stability of all the synthesized hybrids in cell culture medium at 37 °C for up to 24 h using combined HPLC-MS/MS techniques.

As depicted in Figure 2, UDCMe-(1,5)-t-DHA and CDCMe-(1,4)-t-DHA were chemically stable in cell culture medium after 24 h of incubation at 37 °C, whereas CDC-DHA, CDCMe-s-DHA, and CDCMe-(1,5)-t-DHA were significantly less stable. Specifically, CDC-DHA underwent 22% degradation, CDCMe-s-DHA 21%, and CDCMe-(1,5)-t-DHA 31% after 24 h (Figure 2). For the fused hybrids, UDC-DHA and CDC-DHA, conjugated at C24 of the proper BA, stability appears to be primarily determined by the nature of the bile acid (UDC > CDC). In contrast, for conjugation at C3, the nature of the linker seems to play a more significant role. The succinyl (cleavable) linker induces instability in both UDCMe-s-DHA and CDCMe-s-DHA, with a more pronounced effect observed for the UDC-based hybrid (UDC < CDC; 82% vs. 21% degradation, respectively). When the 1,2,3-triazole (non-cleavable) linker is employed, the influence of the BA component appears less critical. Indeed, both (1,4)-triazole hybrids, UDCMe-(1,4)-t-DHA and CDCMe-(1,4)-t-DHA, were stable, whereas within the (1,5)-triazole series, CDCMe-(1,5)-t-DHA was slightly less stable than UDCMe-(1,5)-t-DHA (UDC > CDC) (Figure 2). Overall, these findings indicate that the chemical stability of the hybrids results from a complex interplay between the stereochemistry of the BA and the nature of the linker. Moreover, structural variations, like (1,4)- versus (1,5)-triazole linkages, highlight stability differences that depend on the specific BA employed. The data obtained can suggest that the chemical stability of the hybrids in cell culture medium may play a role in governing the biological performance of BA-DHA hybrids towards HCT116 cancer cells. Indeed, in the case of the fused hybrids UDC-DHA and CDC-DHA, which retain the unconjugated C3- and C7-hydroxyl groups and therefore maintain key structural and physicochemical features of the parent BAs, the higher activity observed for UDC-DHA with respect to CDC-DHA may, at least in part, be attributed to its greater chemical stability (Figure 2). Similarly, for the succinyl-linked hybrids UDCMe-s-DHA and CDCMe-s-DHA, the relatively modest enhancement in activity compared to unconjugated DHA likely reflects their lower chemical stability, once again emphasizing the critical influence of hybrid stability on biological performance (Figure 2). With regard to the click-derived hybrids, the (1,4)-triazole derivatives exhibited higher activity compared to their (1,5)-triazole analogs, most notably within the CDC series (Table 1). Once again, the greater instability of CDCMe-(1,5)-t-DHA, which showed approximately 31% degradation after 24 h incubation in cell culture medium, could at least partially account for its lower activity compared with the more stable UDCMe-(1,5)-t-DHA. CDCMe-(1,4)-t-DHA and UDCMe-(1,4)-t-DHA were found to be the most active hybrids of the series in HCT116 cells. Interestingly, both compounds remained stable in cell culture medium for up to 24 h to a comparable extent. However, CDCMe-(1,4)-t-DHA exhibited a higher improvement in activity than the corresponding UDC-based hybrid with respect to the parent DHA, with DHA/Hybrid IC_50_ ratios of 20 and 15, respectively (Table 1). The remarkable difference in cytotoxicity displayed by the CDCMe-(1,4)-t-DHA hybrid compared to the UDCMe-(1,4)-t-DHA one clearly indicates that the nature of the BA can influence the biological activity of the hybrids. In a previous study using HCT116 cells, we found that CDCA conjugated with the chemotherapeutic drug paclitaxel exhibited a greater ability to cross the plasma membrane than the corresponding UDC–paclitaxel hybrid [37]. In light of this observation, we may hypothesize that a similar mechanism could, at least in part, account for the higher activity of the CDCMe-(1,4)-t-DHA hybrid compared to the UDCMe-(1,4)-t-DHA analog and with respect to parent DHA in HCT116 cells. It is worth noting that the nature of the triazole linker may also contribute to the enhanced activity observed in the click hybrids UDCMe-(1,4)-t-DHA, CDCMe-(1,4)-t-DHA, CDCMe-(1,5)-t-DHA, and UDCMe-(1,5)-t-DHA. Indeed, the triazole ring can be considered a pharmacophore itself, owing to its ability to improve the pharmacological profile of bioactive molecules as a result of its stability under both physiological and enzymatic conditions [63].

In RKO cells, all hybrids exhibited a less pronounced improvement in activity over unconjugated DHA compared with HCT116 cells. Furthermore, in RKO cells, the difference in activity between UDC- and CDC-based hybrids was much less marked than that observed in HCT116 cells. The most active hybrid in RKO cells was UDCMe-(1,5)-t-DHA with IC_50_ = 0.86 μM, corresponding to a 6-fold increased effectiveness with respect to unconjugated DHA. With regard to the click hybrids, unlike in HCT116 cells, the (1,5)-triazole derivatives exhibited higher activity than the (1,4)-triazole analogs in RKO, most notably in the UDC series.

Overall, these findings suggest that the conjugation via click chemistry enhances hybrid activity, while the triazole linker structure modulates this effect in a cell line-specific way.

Notably, neither CDCA nor UDCA, when tested under our experimental conditions, exhibited significant cytotoxic activity against HCT116 or RKO cell lines, thereby confirming the concentration-dependent nature of this effect [56].

DHA has previously been reported to induce cell cycle arrest in the G0/G1 or G2/M phases in various cancer cells [10,39,40]. In the present study, we observed that a 36 h treatment with all BA-DHA hybrids at 5 μM concentration significantly increased the G0/G1 population in a superior (HCT116 cells) or comparable manner (RKO cells) to that of unconjugated DHA at 50 μM concentration. A marked reduction in the S phase was also observed, suggesting that these compounds may impede cell proliferation in both HCT116 and RKO cells. It is worth noting that the increase in the G0/G1 population was more pronounced in HCT116 cells than in RKO cells, and that the click hybrids UDCMe-(1,5)-t-DHA and CDCMe-(1,5)-t-DHA were more effective than the other hybrids in reducing the S phase in HCT116 cells. We also observed that DHA induced a significant increase in the G2/M population, while the BA-DHA hybrids caused a decrease in the G2/M population after 36 h of treatment (Figure 4). It is known, indeed, that DHA can induce both G0/G1 and G2/M cell cycle arrest in HCT116 cells by broadly interfering with multiple checkpoints through oxidative and endoplasmic reticulum stress induction [64,65,66], whereas there is evidence that DHA derivatives, such as ruthenium–DHA complexes, can selectively arrest cells in the G0/G1 phase [64], likely due to the targeted inhibition of early cycle regulators like cyclin D/CDK4 without triggering DNA damage responses.

Results shown in Figure 5A indicate that DHA at 50 μM concentration and the click hybrids (namely UDCMe-(1,4)-t-DHA, UDCMe-(1,5)-t-DHA, CDCMe-(1,4)-t-DHA, and CDCMe-(1,5)-t-DHA) at 5 μM concentration induced apoptosis in HCT116 cells to a comparable extent. These findings demonstrate that both (1,4)- and (1,5)-triazole hybrids, based on either UDCA or CDCA, are capable of inducing apoptosis at concentrations that are an order of magnitude lower than DHA in HCT116 cells. In contrast, in RKO cells, all tested hybrids induced a lower apoptosis rate compared to DHA, taking into account that they were used at a 10-fold lower concentration than DHA.

To further assess the biological impact of the hybrids beyond their cytostatic potential, the migration and invasion abilities of HCT116 cells were evaluated by administering DHA at a concentration of 50 μM and the corresponding DHA-BA hybrids at 5 μM. As shown in Figure 6A,B, respectively, both cell migration and invasion were markedly inhibited in cells treated with DHA as well as by BA-DHA hybrids compared with controls. Notably, the click-derived UDCMe-(1,5)-t-DHA, exhibited the most pronounced inhibitory activity on cell migration, as shown also by slope values (Figure 6A), as well as on HCT116 cell invasion (Figure 6B), demonstrating a 3-fold higher effect than DHA after 48 h, even if used with 10-fold less concentration.

The ability of DHA to suppress angiogenesis has been documented in several experimental models, although its precise molecular mechanism remains incompletely defined [67]. In light of the findings presented above, subsequent investigations on the potential antiangiogenic effects were focused on the click hybrids UDCMe-(1,4)-t-DHA, UDCMe-(1,5)-t-DHA, CDCMe-(1,4)-t-DHA and CDCMe-(1,5)-t-DHA with respect to DHA alone on human umbilical vein endothelial cells (HUVECs), with a specific concern on cell proliferation and on the modulation of VEGFR-2 (Vascular Endothelial Growth Factor Receptor-2) expression, involved in one of the major pathways driving endothelial proliferation. The VEGFR-2 is a transmembrane tyrosine kinase that mediates most of the biological effects of VEGF-A, including endothelial cell proliferation, migration, and new capillary formation. Upon ligand binding, VEGFR-2 undergoes dimerization and autophosphorylation, triggering intracellular cascades such as the PI3K/AKT and MAPK/ERK pathways, which promote cell survival and vascular morphogenesis. Therefore, inhibition of VEGFR-2 signaling represents a major therapeutic target to block pathological angiogenesis, particularly in tumor growth and metastasis [68].

We selected HUVECs as our experimental model as they represent a well-established and reproducible system to study human endothelial cell behavior. HUVECs retain the typical morphology, receptor profile, and angiogenic responsiveness of vascular endothelium, making them ideal for investigating compounds that modulate endothelial activation and new vessel formation [69].

Our results show that, in the vascular compartment, DHA and the click hybrids exert similar effects, although the latter were used at a 10-fold lower concentration than DHA. These findings confirm the previously described mechanisms underlying the antitumor activity of DHA, namely, inhibition of cell proliferation and downregulation of VEGFR-2 in endothelial cells, as reported by Dong et al. [70], and further highlight the effectiveness of click chemistry in enhancing DHA’s ability to counteract multiple processes involved in tumor progression (Figure 7 and Figure 8).

## 5. Conclusions

The present findings demonstrate that conjugation of DHA with UDCA and CDCA considerably enhances its in vitro biological activity in both colorectal cancer (CRC) cell lines tested.

The synergistic interplay between the linker and conjugation site produced considerably active click-derived hybrids conjugated at the C3 of both UDCA and CDCA, which demonstrated pronounced activity in both cell lines tested. In particular, CDCMe-(1,4)-t-DHA showed the greatest potency in HCT116 cells, whereas UDCMe-(1,5)-t-DHA was the most effective hybrid in RKO cells.

The click-derived hybrids from both the UDCA- and CDCA-based series were demonstrated to be the most effective inhibitors of cell migration and invasion in HCT116 cells, with UDCMe-(1,5)-t-DHA exerting the strongest anti-migratory activity and outperforming DHA alone. Notably, the (1,5)-triazole derivatives UDCMe-(1,5)-t-DHA and CDCMe-(1,5)-t-DHA suppressed cell invasion more effectively than DHA. Furthermore, all click hybrids were found to strongly inhibit endothelial cells’ proliferation and down-modulate their surface expression of VEGFR-2.

Taken together, these results indicate that the click hybrids possess enhanced anticancer activity and antimetastatic potential, achieving comparable or even superior activity with respect to the parent DHA at markedly lower concentrations and highlight their potential as lead candidates for colorectal cancer (CRC) therapy. Moreover, the SAR analysis presented in this study provides valuable insights into the rational design of BA-based hybrids and supports the development of next-generation BA conjugates with improved biological properties.

## Figures and Tables

**Table 1:** IC_50_ values ± SEM (standard error of the mean) of DHA and BA-DHA hybrids in HCT116 and RKO cells.

	**HCT116**		**RKO**	
**Compound**	**IC_50_ (μM)**	DHA/Hybrid (*)	**IC_50_ (μM)**	DHA/Hybrid (*)
DHA	10.355 ± 1.219	-	5.538 ± 0.463	-
UDC-DHA	0.876 ± 0.101	12	1.875 ± 0.446	3
UDCMe-s-DHA	2.157 ± 0.227	5	1.643 ± 0.220	3
UDCMe-(1,4)-t-DHA	0.692 ± 0.141	15	1.478 ± 0.268	4
UDCMe-(1,5)-t-DHA	0.727 ± 0.274	14	0.856 ± 0.166	6
CDC-DHA	1.332 ± 0.175	8	2.015 ± 0.372	3
CDCMe-s-DHA	2.233 ± 0.391	5	2.328 ± 0.297	2
CDCMe-(1,4)-t-DHA	0.523 ± 0.096	20	1.539 ± 0.231	4
CDCMe-(1,5)-t-DHA	0.819 ± 0.369	13	1.056 ± 0.159	5

(*) The DHA/Hybrid value was calculated as the ratio of the IC_50_ of DHA and the hybrid.

## Data Availability

The original contributions presented in this study are included in the article/Supplementary Materials. Further inquiries can be directed to the corresponding authors.

## Supplementary Materials

The following supporting information can be downloaded at https://www.mdpi.com/article/10.3390/biom16010177/s1, Figure S1: ^1^H-NMR (400 MHz, CDCl_3_) spectrum of UDCMe-(1,4)-t-DHA; Figure S2: ^13^C-NMR (101 MHz, CDCl_3_) spectrum of UDCMe-(1,4)-t-DHA; Figure S3: MS (ESI+) spectrum of UDCMe-(1,4)-t-DHA; Figure S4: ^1^H-NMR (400 MHz, CDCl_3_) spectrum of CDCMe-(1,4)-t-DHA; Figure S5: ^13^C-NMR (101 MHz, CDCl_3_) spectrum of CDCMe-(1,4)-t-DHA; Figure S6: MS (ESI+) spectrum of CDCMe-(1,4)-t-DHA; Figure S7: ^1^H-NMR (400 MHz, CDCl_3_) spectrum of UDCMe-(1,5)-t-DHA; Figure S8: ^13^C-NMR (101 MHz, CDCl_3_) spectrum of UDCMe-(1,5)-t-DHA; Figure S9: MS (ESI+) spectrum of UDCMe-(1,5)-t-DHA; Figure S10: ^1^H-NMR (400 MHz, CDCl_3_) spectrum of CDCMe-(1,5)-t-DHA; Figure S11: ^13^C-NMR (101 MHz, CDCl_3_) spectrum of CDCMe-(1,5)-t-DHA; Figure S12: MS (ESI+) spectrum of CDCMe-(1,5)-t-DHA; Figure S13: Effects of DHA on HCT116 and RKO viable cells’ number; Figure S14: Effects of DHA and DHA hybrids on HCT116 and RKO metabolic activity; Figure S15: Cytostatic effects of UDC-DHA, UDCMe-s-DHA, CDC-DHA, CDCMe-s-DHA and BAs on HCT116 and on RKO cell cycle; Table S1: Significance of the MTT data analysis for each hybrid vs. DHA in the HCT116 and RKO cell lines.

## Abbreviations

The following abbreviations are used in this manuscript:

CRC	Colorectal cancer
NP	Natural product
DHA	Dihydroartemisinin
BA	Bile acid
CDCA	Chenodeoxycholic bile acid
UDCA	Ursodeoxycholic bile acid
SAR	Structure–activity relationship
MH	Molecular hybridization
FXR	Farnesoid X Receptor
