# Supplementary material for: Biological Evaluation and SAR Exploration of Bile Acid–Dihydroartemisinin Hybrids as Potential Anticancer Agents for Colorectal Cancer
Source: Biomolecules. 2026 Jan 22;16(1):177. doi: 10.3390/biom16010177 (PMC12838976; doi:10.3390/biom16010177)
Supplement: Supplementary file 1 [file biomolecules-16-00177-s001.zip › biomolecules-4063768-supplementary.pdf]

Biological Evaluation and SAR Exploration of Bile Acid-Dihydroartemisinin Hybrids as Potential Anticancer Agents for Colorectal Cancer

Daniela Perrone [1,†], Elisabetta Melloni [2,†], Lorenzo Gnudi [1], Fabio Casciano [3], Elena Pozza [4], Francesca Bompan [1], Paola Secchiero [2], Elena Marchesi [5,*] and Maria Luisa Navacchia [6,*]

[1] *Department of Environmental and Prevention Sciences, University of Ferrara, 44121 Ferrara, Italy;* prd@unife.it (D.P.); lorenzo.gnudi@unife.it (L.G.); francesca.bompan@unife.it (F.B.)
[2] *Department of Translational Medicine and LTTA Centre, University of Ferrara, 44121 Ferrara, Italy;* elisabetta.melloni@unife.it (E.M.); paola.secchiero@unife.it (P.S.)
[3] *Department of Environmental and Prevention Sciences and LTTA Centre, University of Ferrara, 44121 Ferrara, Italy*
[4] *Department of Translational Medicine, University of Ferrara, 44121 Ferrara, Italy;* pzzlne1@unife.it (E.P.)
[5] *Department of Chemical, Pharmaceutical and Agricultural Sciences, University of Ferrara, 44121 Ferrara, Italy;*
[6] *Institute for Organic Synthesis and Photoreactivity (ISOF), National Research Council of Italy (CNR), 40129 Bologna, Italy*
* Correspondence: mrclne@unife.it (E.M.); marialuisa.navacchia@isof.cnr.it (M.L.N.); .
† These authors equally contributed to the work

Supporting information

Contents

	Page
Figure S1: ^{1}H-NMR (400 MHz, CDCl3) spectrum of UDCMe-(1,4)-t-DHA	3
Figure S2: ^{13}C-NMR (101 MHz, CDCl3) spectrum of UDCMe-(1,4)-t-DHA	4
Figure S3: MS (ESI+) spectrum of UDCMe-(1,4)-t-DHA	5
Figure S4: ^{1}H-NMR (400 MHz, CDCl3) spectrum of CDCMe-(1,4)-t-DHA	6
Figure S5: ^{13}C-NMR (101 MHz, CDCl3) spectrum of CDCMe-(1,4)-t-DHA	7
Figure S6: MS (ESI+) spectrum of CDCMe-(1,4)-t-DHA	8

Figure S7: ^{1}H-NMR (400 MHz, CDCl3) spectrum of UDCMe-(1,5)-t-DHA 9

Figure S8: ^{13}C-NMR (101 MHz, CDCl3) spectrum of UDCMe-(1,5)-t-DHA 10

Figure S9: MS (ESI+) spectrum of UDCMe-(1,5)-t-DHA 11

Figure S10: ^{1}H-NMR (400 MHz, CDCl3) spectrum of CDCMe-(1,5)-t-DHA 12

Figure S11: ^{13}C-NMR (101 MHz, CDCl3) spectrum of CDCMe-(1,5)-t-DHA 13

Figure S12: MS (ESI+) spectrum of CDCMe-(1,5)-t-DHA 14

Figure S13: Effects of DHA on HCT116 and RKO viable cells' number 15

Figure S14: Effects of DHA and DHA hybrids on HCT116 and RKO metabolic activity 16

Table S1: Significance of the MTT data analysis for each hybrid vs. DHA in the HCT116 and RKO cell lines 17

Figure S15: Cytostatic effects of UDC-DHA, UDCMe-s-DHA, CDC-DHA, CDCMe-s-DHA and BAs on HCT116 and on RKO cell cycle 18

Figure S1: ^{1}H-NMR (400 MHz, $CDCl_3$) spectrum of UDCMe-(1,4)-t-DHA

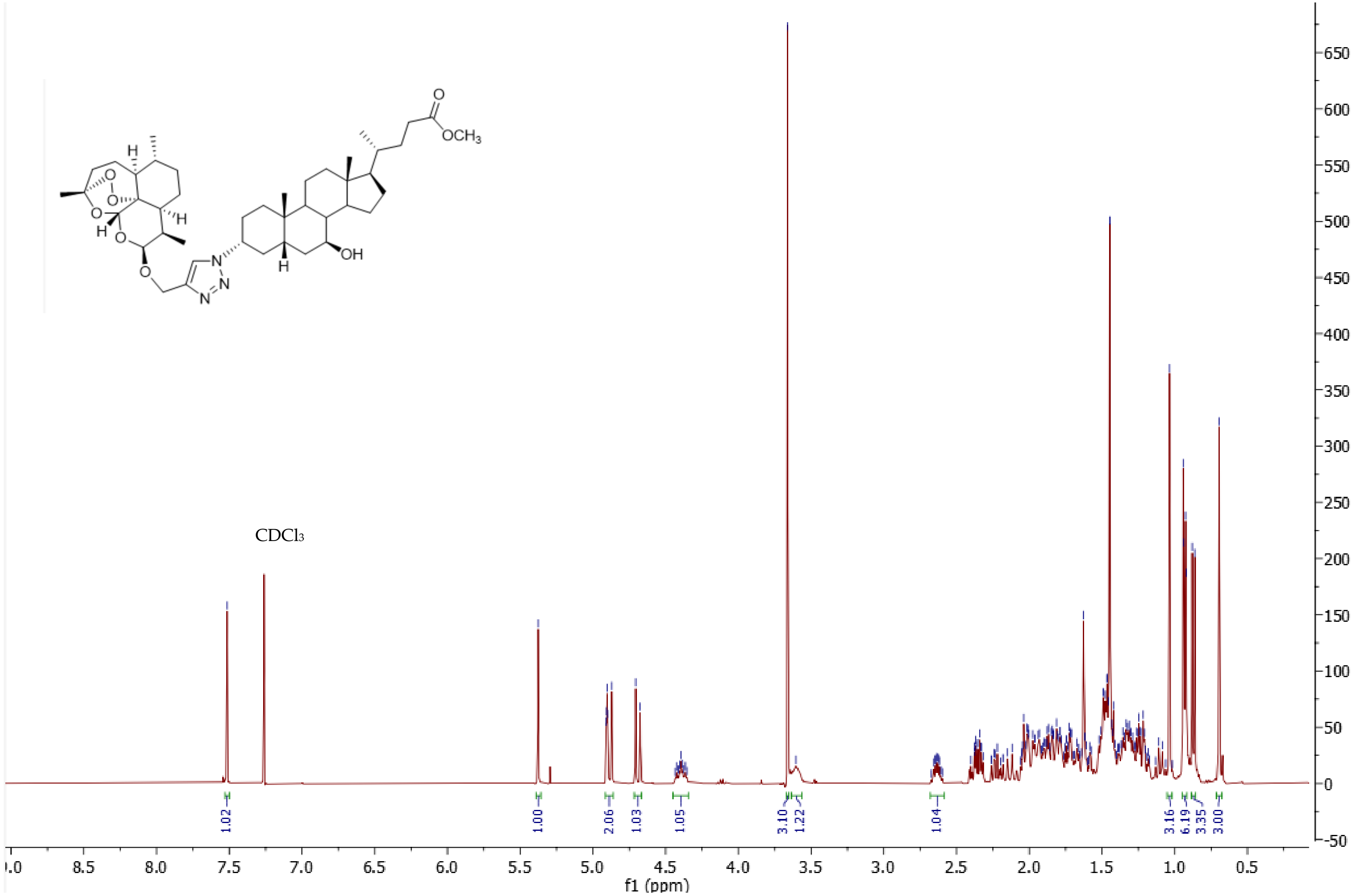

Figure S2: ^{13}C-NMR (101 MHz, $CDCl_3$) spectrum of UDCMe-(1,4)-t-DHA

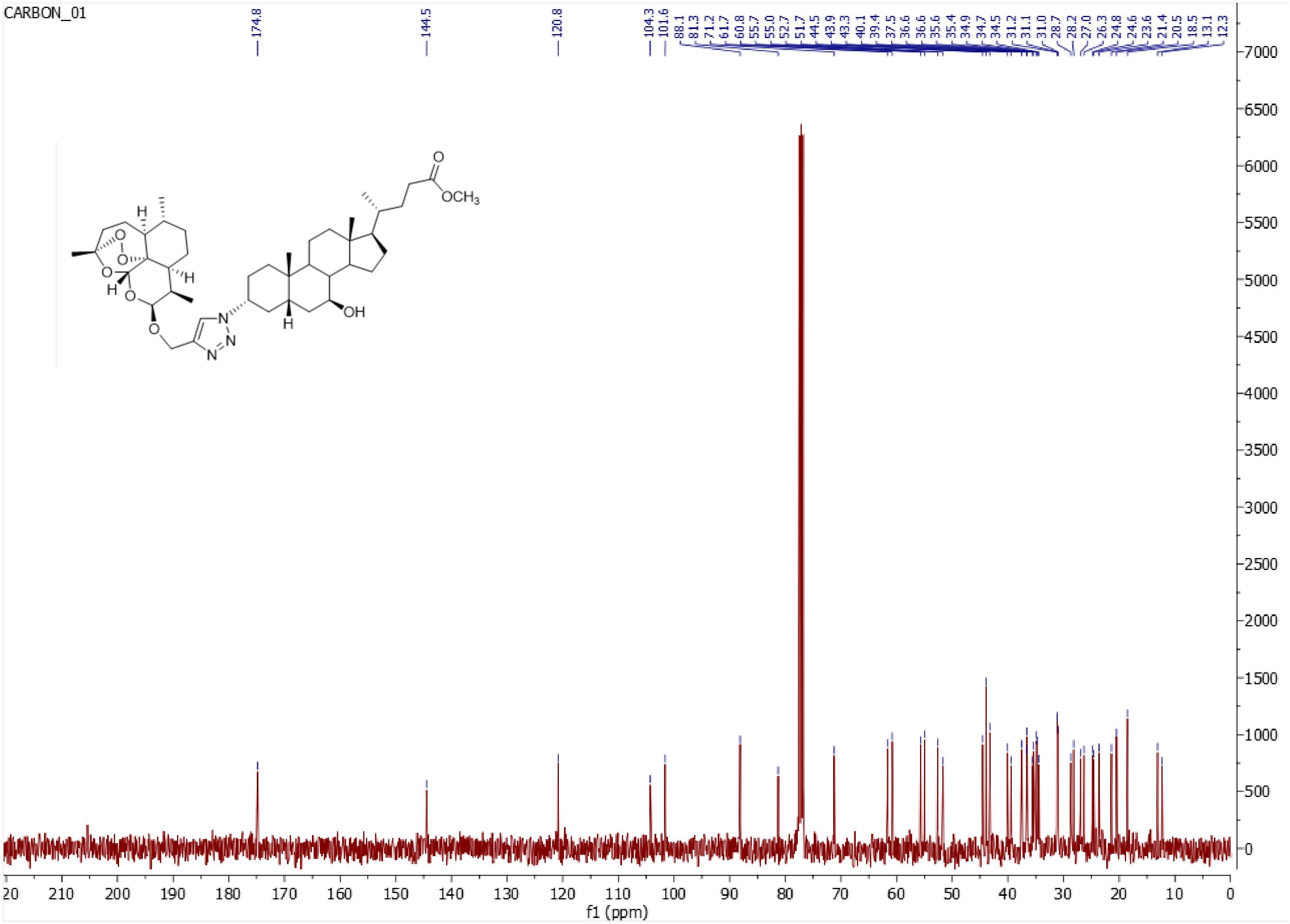

Figure S3: MS (ESI+) **spectrum of UDCMe-(1,4)-t-DHA**

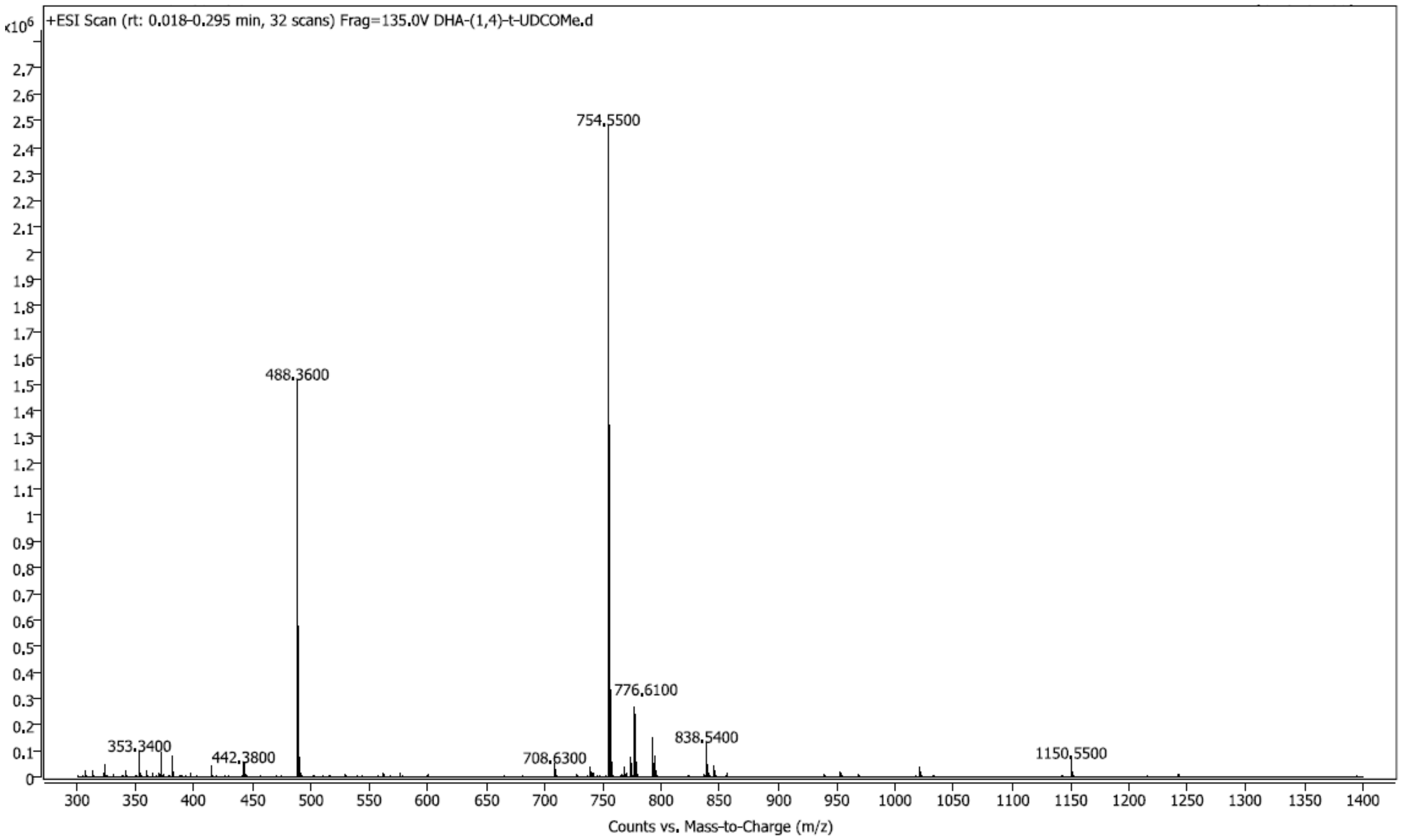

Figure S4: ^{1}H-NMR (400 MHz, $CDCl_3$) spectrum of CDCMe-(1,4)-t-DHA

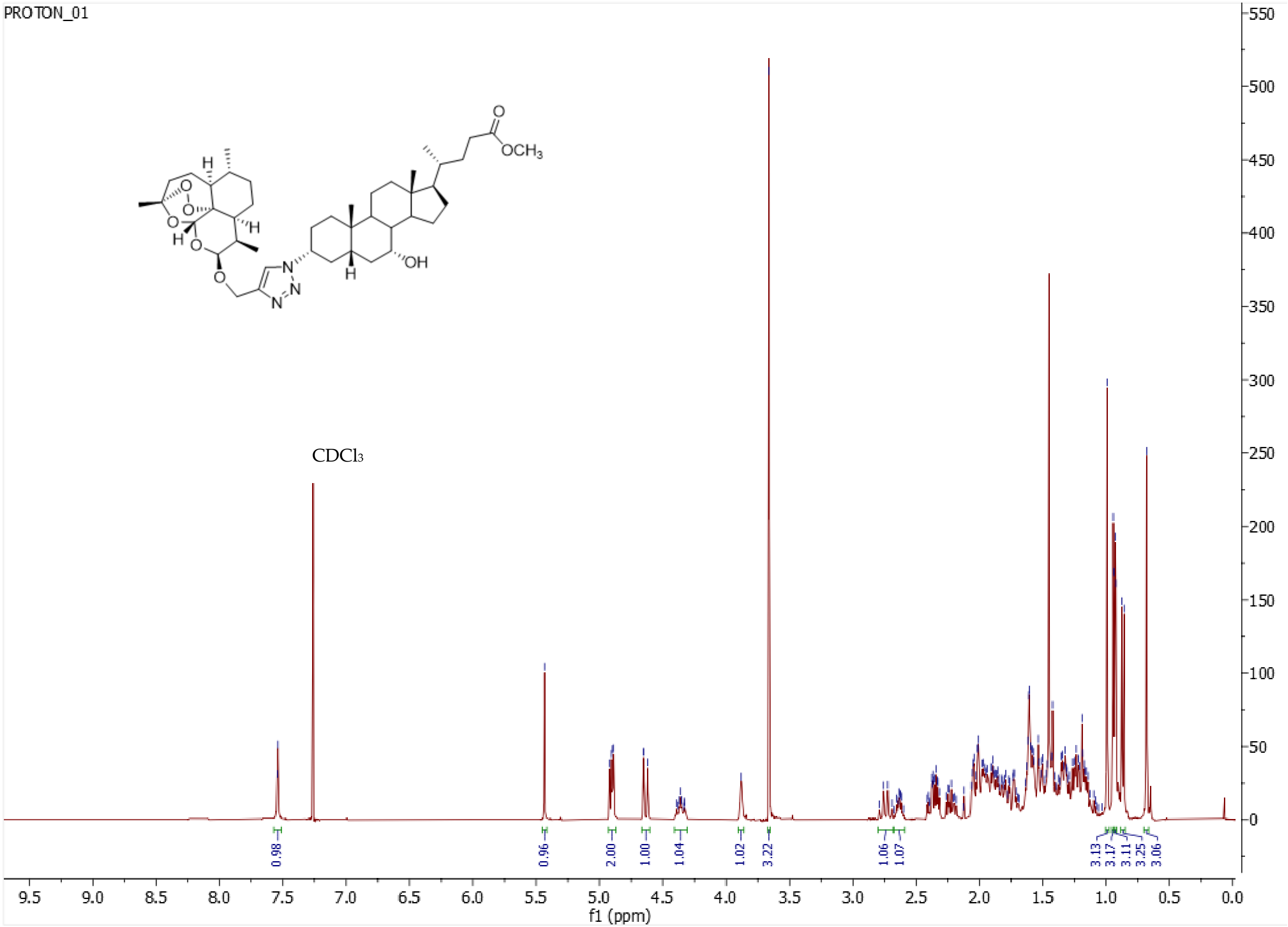

Figure S5: ^{13}C-NMR (101 MHz, $CDCl_3$) spectrum of CDCMe-(1,4)-t-DHA

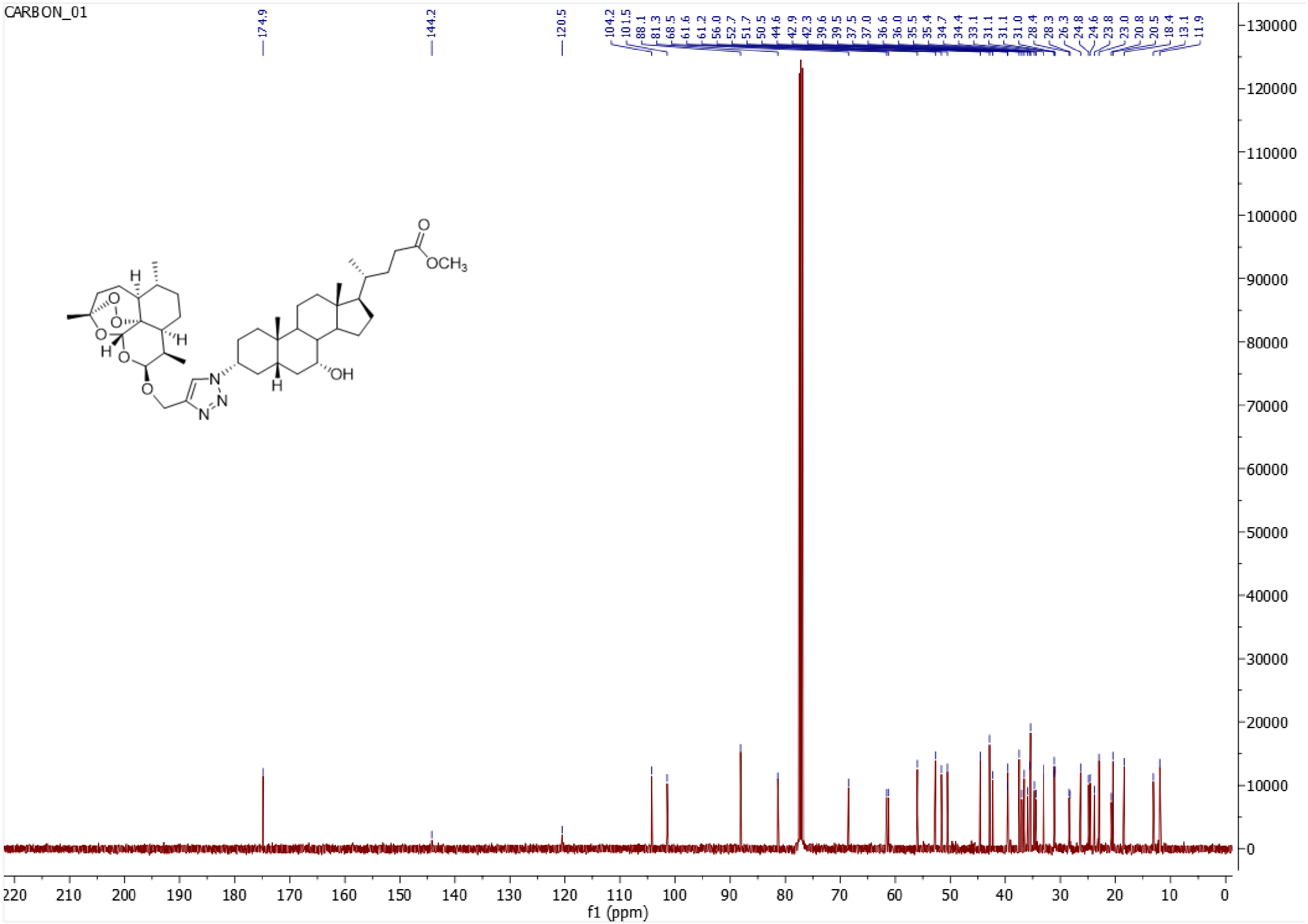

Figure S6: MS (ESI+) spectrum of CDCMe-(1,4)-t-DHA

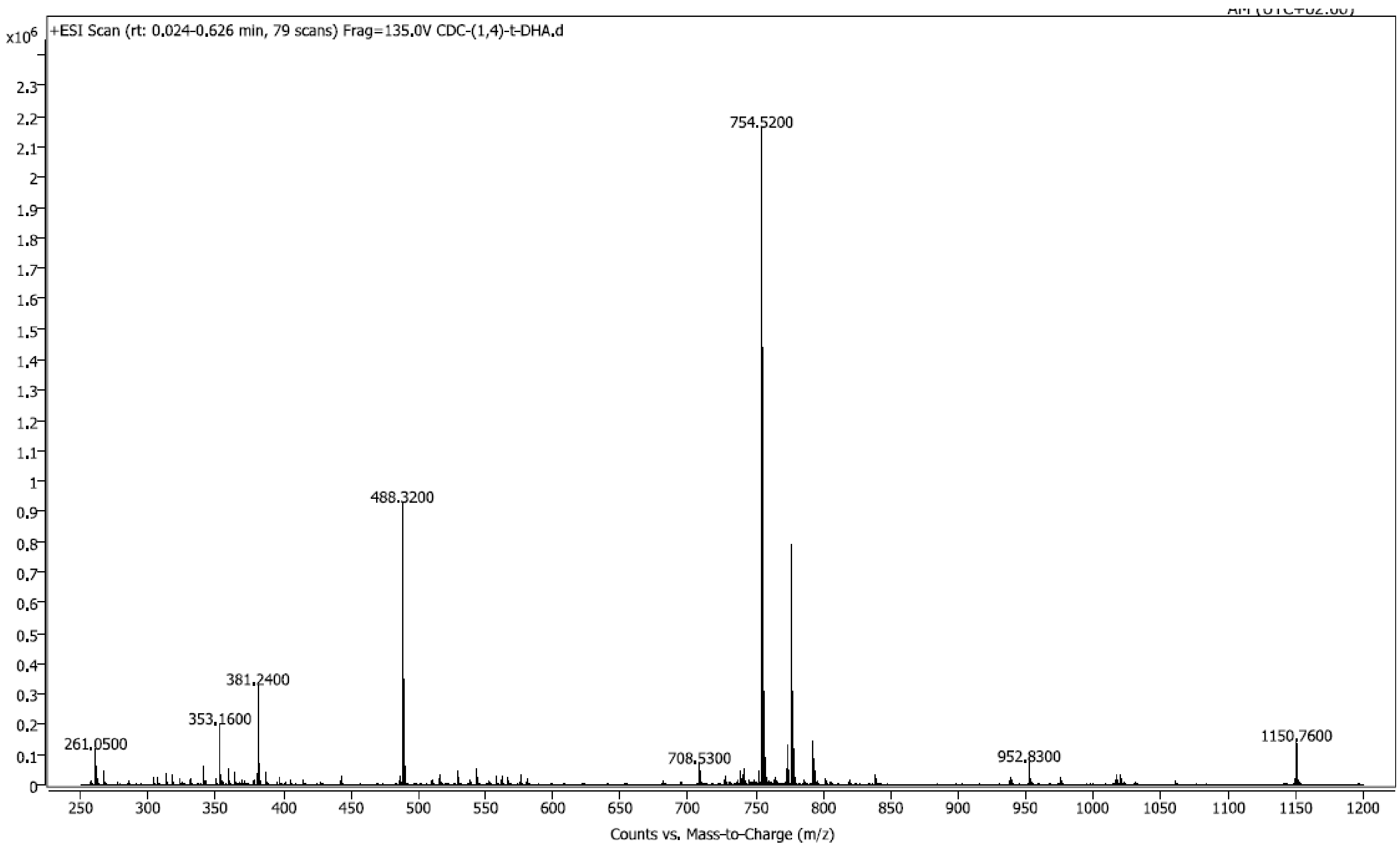

Figure S7: ^{1}H-NMR (400 MHz, $CDCl_3$) spectrum of UDCMe-(1,5)-t-DHA

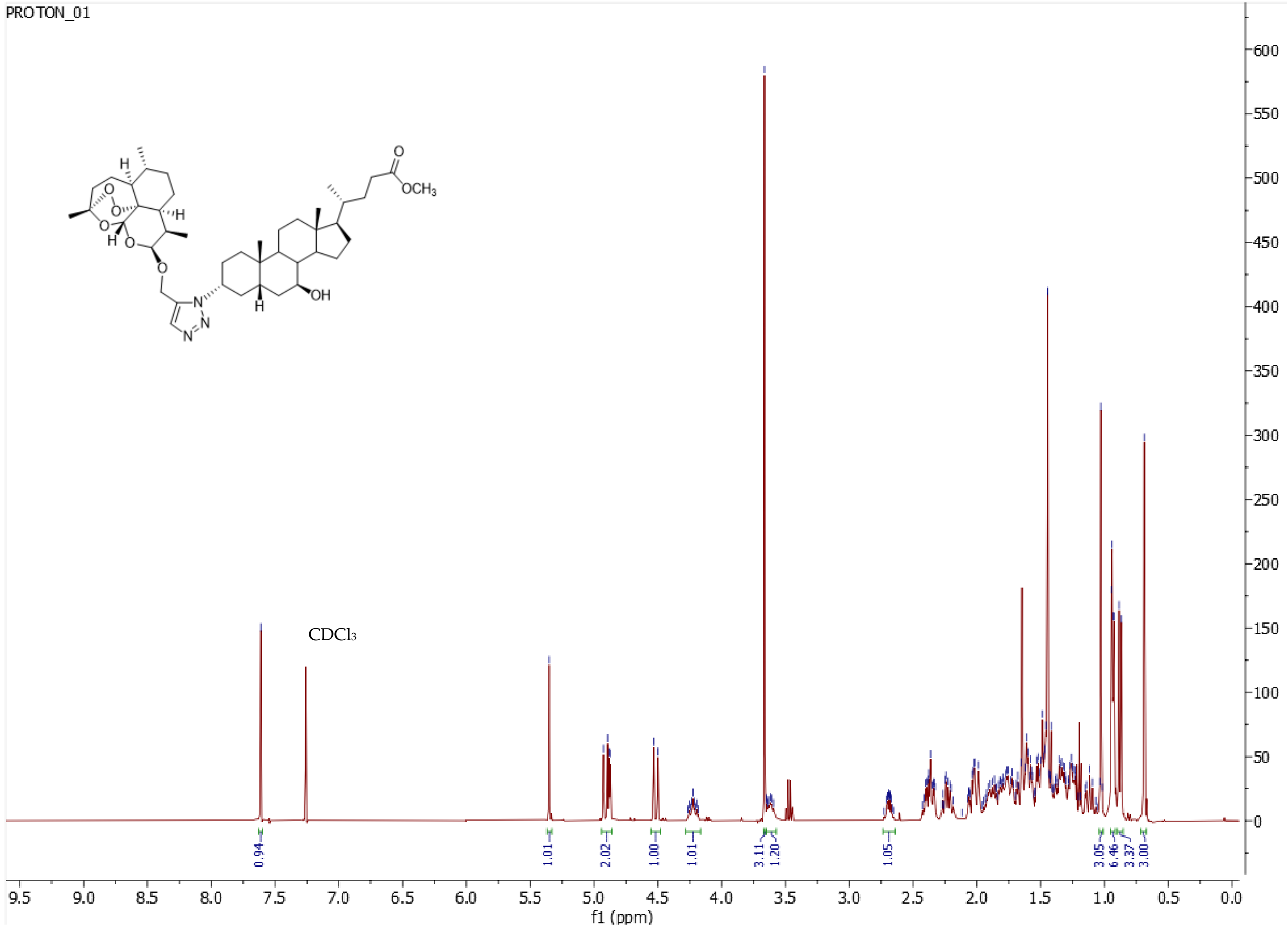

Figure S8: ^{13}C-NMR (101 MHz, $CDCl_3$) spectrum of UDCMe-(1,5)-t-DHA

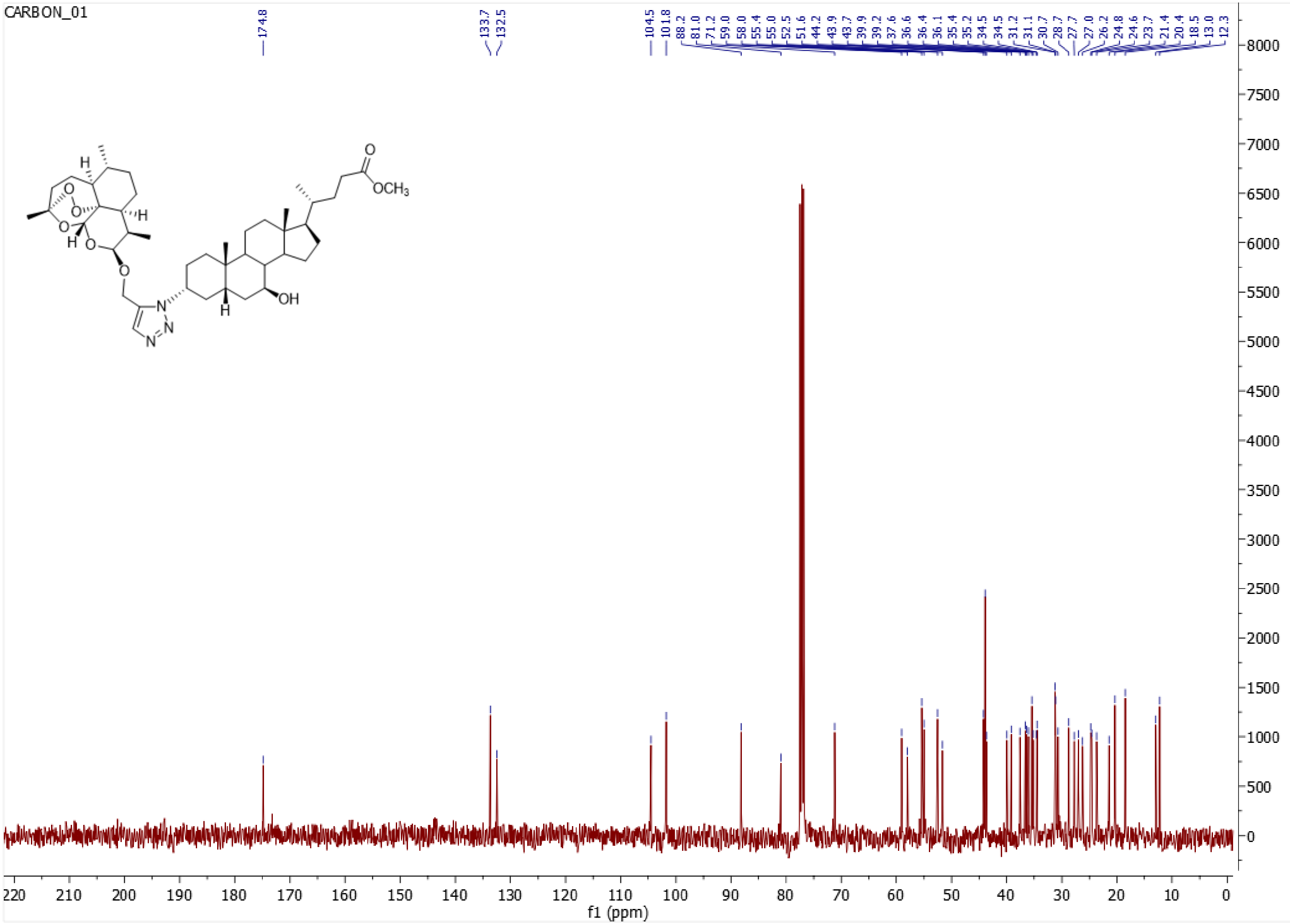

Figure S9: MS (ESI+) spectrum of UDCMe-(1,5)-t-DHA

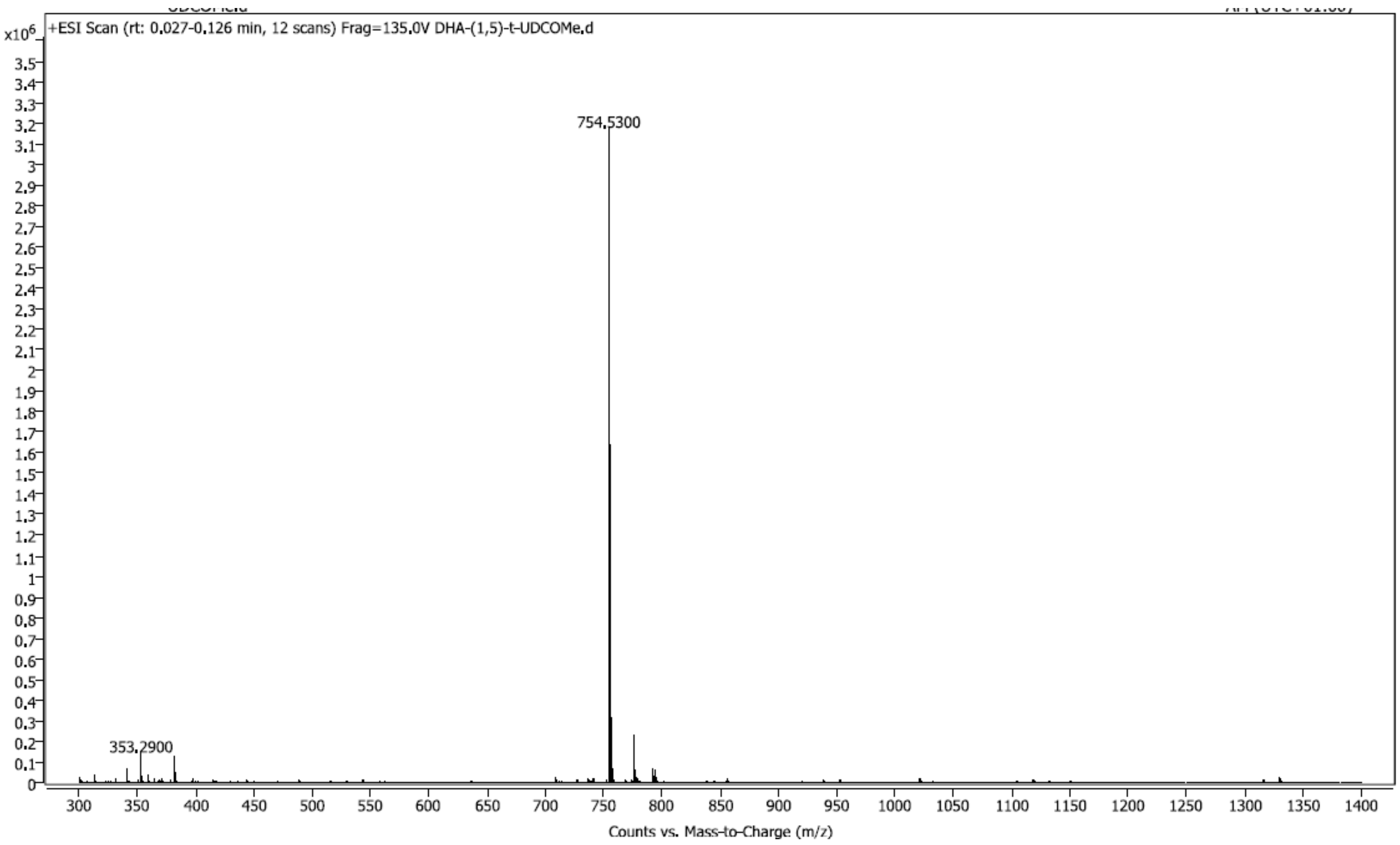

Figure S10: ^{1}H-NMR (400 MHz, $CDCl_3$) spectrum of CDCMe-(1,5)-t-DHA

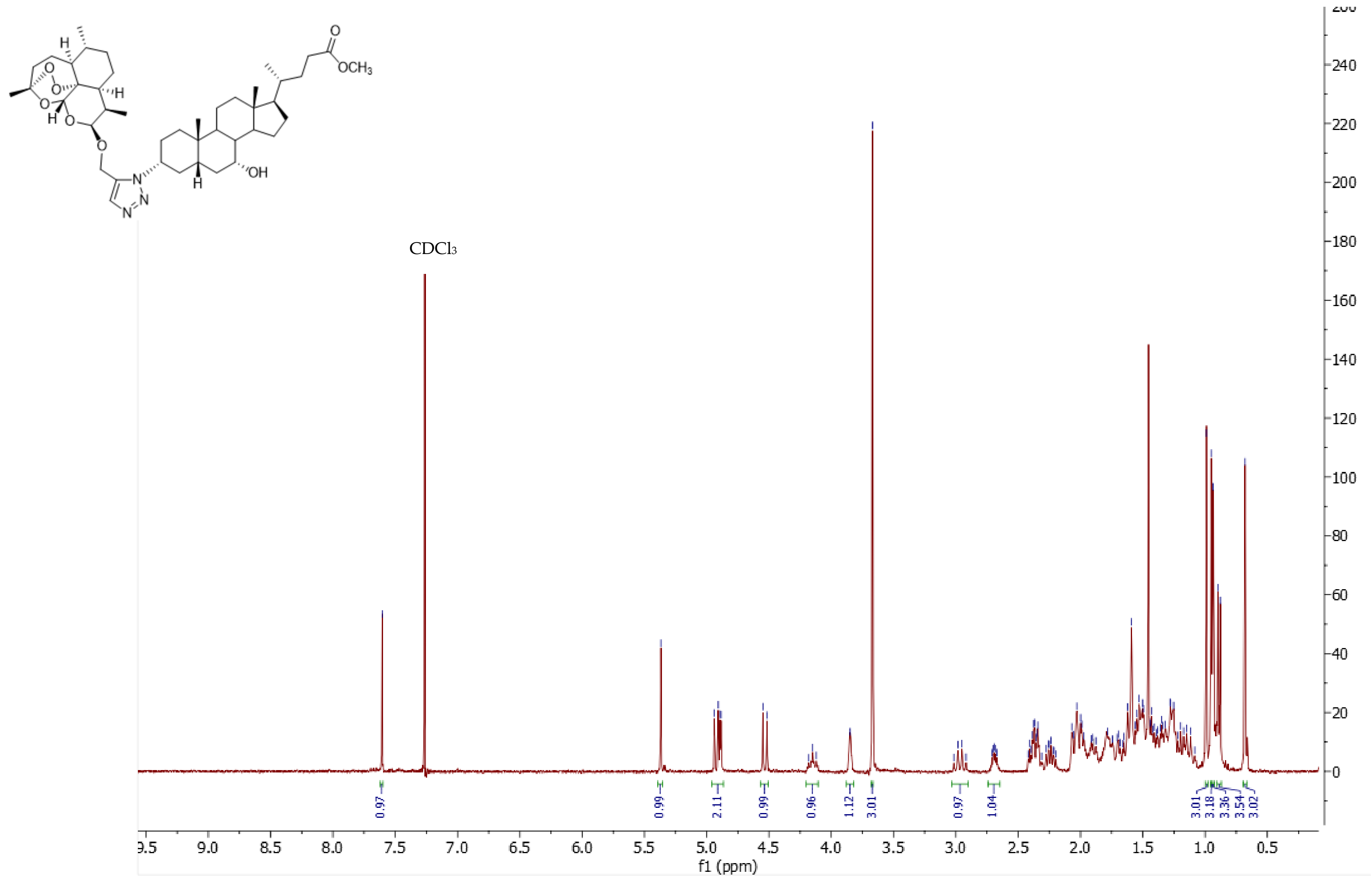

Figure S11: ^{13}C-NMR (101 MHz, $CDCl_3$) spectrum of CDCMe-(1,5)-t-DHA

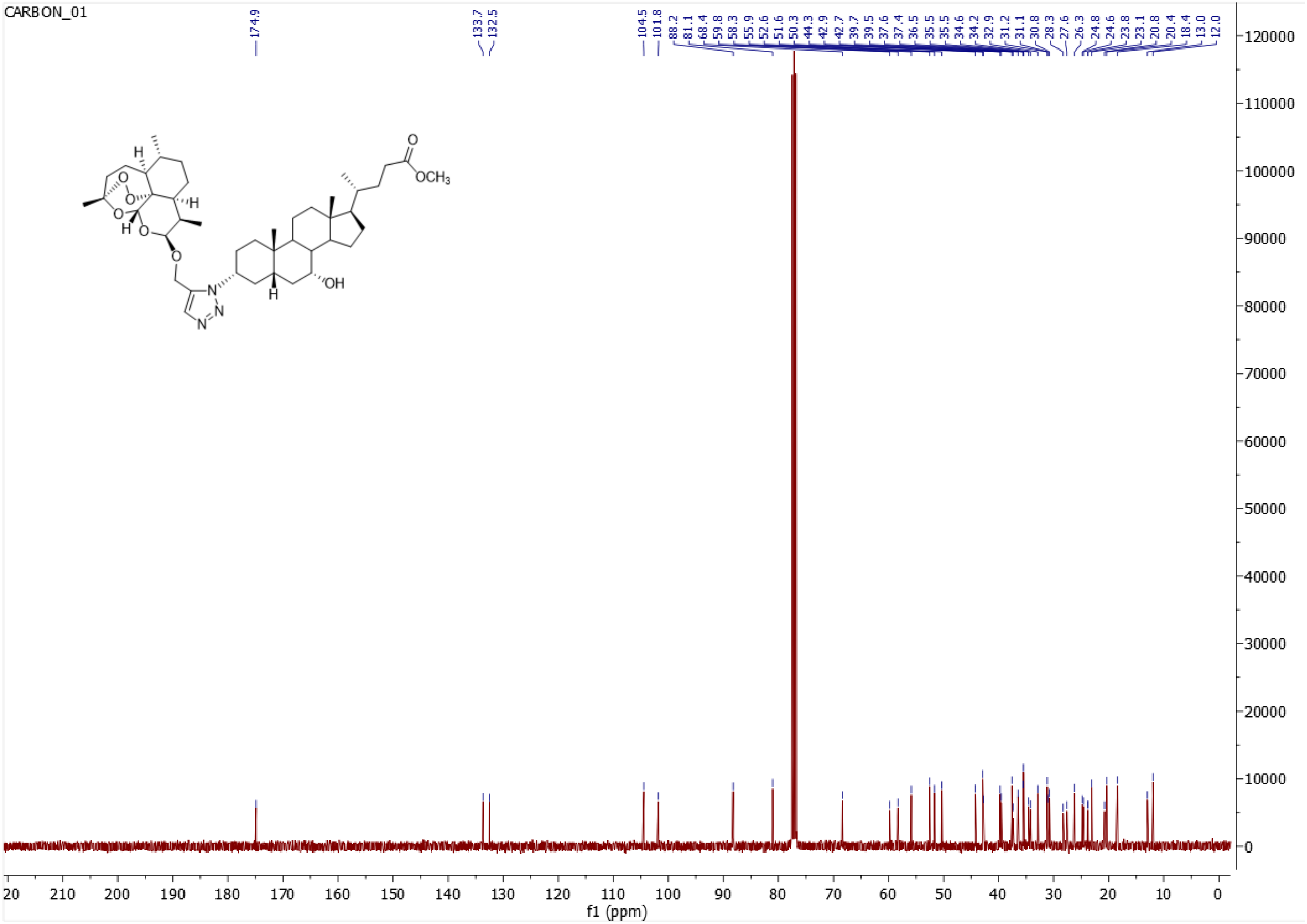

Figure S12: MS (ESI+) spectrum of CDCMe-(1,5)-t-DHA

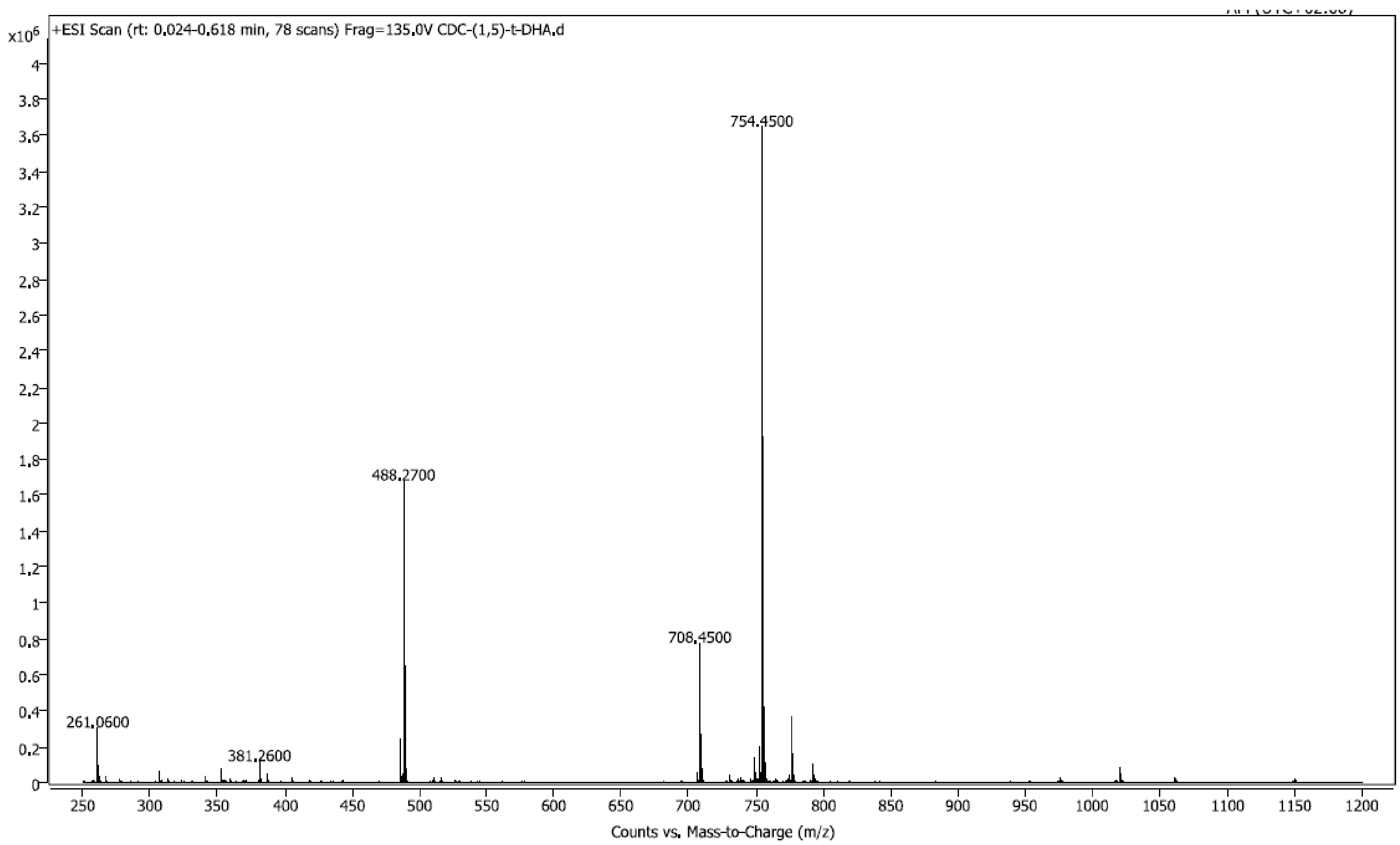

Figure S13

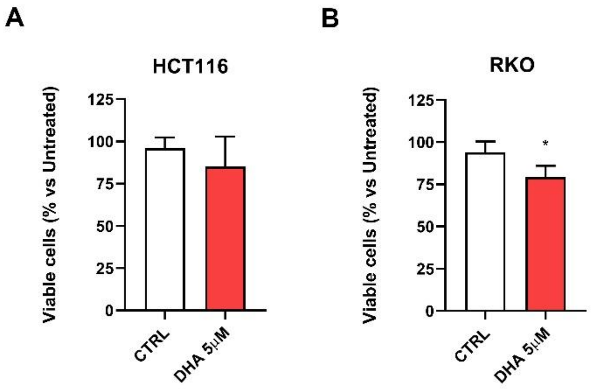

Figure S13: Effects of DHA on HCT116 and RKO viable cells' number. Cell viability was evaluated in HCT116 cells (A) and RKO cells (B) treated for 72 h with 5 μM DHA. Results are reported as mean ± SD of at least three independent experiments. Statistical analysis was performed by Student's t-test. * $p < 0.05$ with respect to control (CTRL).

Figure S14

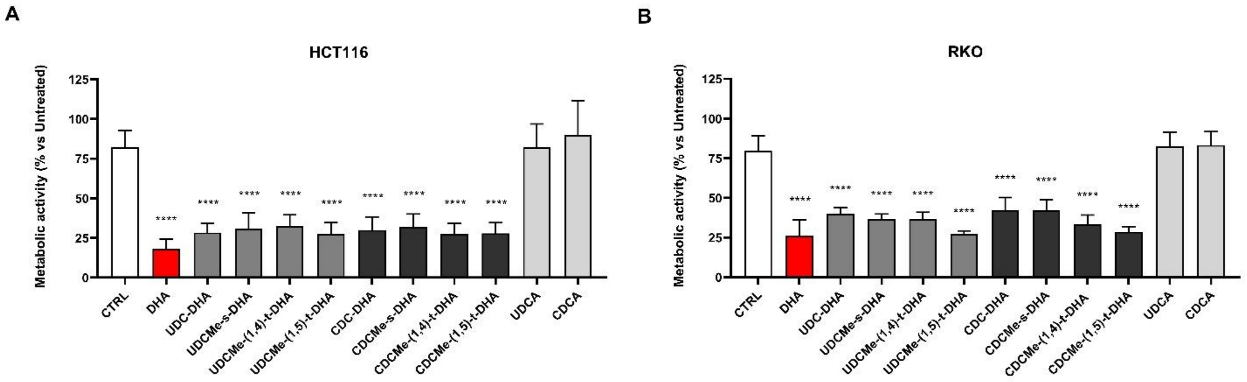

Figure S14: Effects of DHA and DHA hybrids on HCT116 and RKO metabolic activity. Metabolic activity was evaluated by MTT test in both HCT116 (A) and RKO cells (B) treated for 72 h with 50 μM DHA and 5 μM DHA hybrids or BAs alone. Results are reported as mean ± SD of at least three independent experiments. Statistical analysis was performed using one-way ANOVA followed by the Bonferroni post-hoc test. **** $p < 0.0001$ with respect to control (CTRL).

Table S1: Significance of the MTT data analysis for each hybrid vs DHA in the HCT116 and RKO cell lines. Significance p-values were calculated by performing one-way ANOVA test followed by Bonferroni's post-hoc corrections between metabolic activity values of each hybrid vs DHA.

	**HCT116**	**RKO**
Compound	*Significance (p value)*	*Significance (p value)*
**UDC-DHA**	0.6739	0.0114
**UDCMe-s-DHA**	0.4254	0.2132
**UDCMe-(1,4)-t-DHA**	0.1273	0.0865
**UDCMe-(1,5)-t-DHA**	>0.9999	>0.9999
**CDC-DHA**	0.3691	0.0019
**CDCMe-s-DHA**	0.2488	0.0108
**CDCMe-(1,4)-t-DHA**	0.9577	0.5769
**CDCMe-(1,5)-t-DHA**	>0.9999	>0.9999

Figure S15

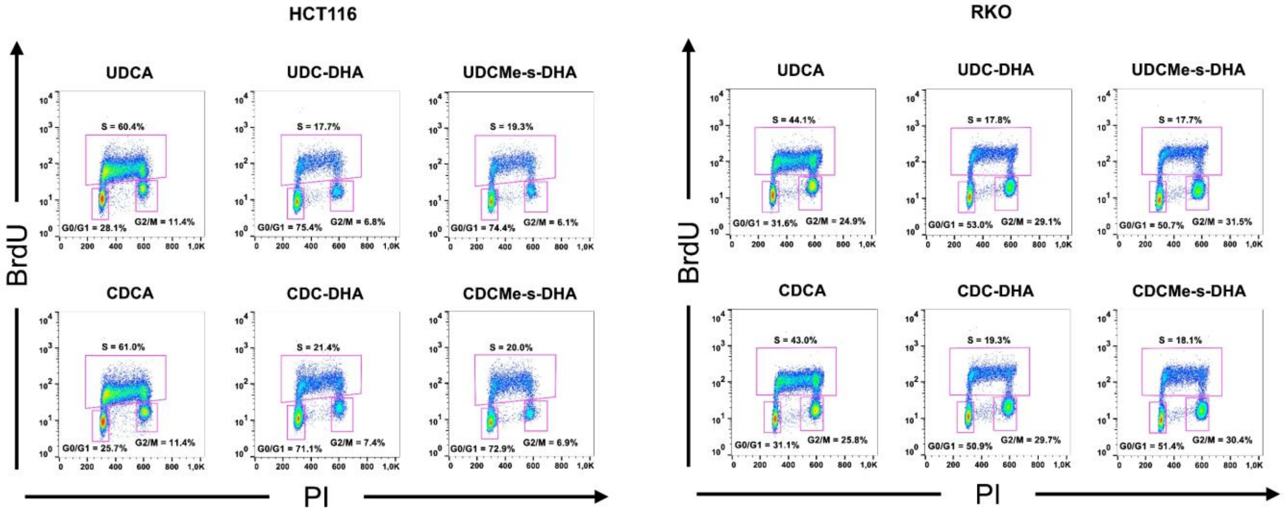

Figure S15: Cytostatic effects of UDC-DHA, UDCMe-s-DHA, CDC-DHA, CDCMe-s-DHA and BAs on HCT116 and on RKO cell cycle. Representative cytometric panel of HCT116 and RKO cell cycle after treatment for 36 h with UDCA, CDCA, UDC-DHA, CDC-DHA, UDCMe-s-DHA and CDCMe-s-DHA (5 μM).
